# *MyStrengths*, a Strengths-Focused Mobile Health Tool: Participatory Design and Development

**DOI:** 10.2196/18049

**Published:** 2020-07-24

**Authors:** Stian Jessen, Jelena Mirkovic, Lise Solberg Nes

**Affiliations:** 1 Department of Digital Health Research Division of Medicine Oslo University Hospital Oslo Norway; 2 Institute of Clinical Medicine Faculty of Medicine University of Oslo Oslo Norway

**Keywords:** mHealth, participatory design, personal strengths, gameful design, gamification, iterative development, positive approach, co-design, user engagement, mobile phone, chronic care, self-management

## Abstract

**Background:**

People living with chronic illnesses are an increasingly large group. Research indicates that care and self-management should not only focus on the illness and problem-oriented aspects of these individuals’ lives but also support them in recognizing and leveraging their personal strengths in daily life.

**Objective:**

This paper presents the design and developmental process of *MyStrengths*, a mobile health (mHealth) app designed to help its users (people with chronic conditions) both find and make use of their personal strengths in their daily lives. Through 4 consecutive phases, this paper presents participant- and researcher-driven activities, discussions regarding design, and development of both the *MyStrengths* app and its content.

**Methods:**

During the 4 phases, we used a range of methods and activities, including (1) an idea-generating workshop aimed at creating ideas for strengths-supporting features with different stakeholders, including patients, caregivers, relatives, and designers (N=35); (2) research seminars with an international group of experts (N=6), in which the concept, theoretical background, and design ideas for the app were discussed; (3) a series of co-design workshops with people in the user group (N=22) aiming to create ideas for how to, in an engaging manner, design the app; and (4) in 4 developmental iterations, the app was evaluated by people in the user group (N=13). Content and strengths exercises were worked on and honed by the research team, the expert groups, and our internal editorial team during the entire developmental process.

**Results:**

The first phase found a wide range of stakeholder requirements to, and ideas for, strengths-focused mHealth apps. From reviewing literature during the second phase, we found a dearth of research on personal strengths with respect to people living with chronic illnesses. Activities during the third phase creatively provided numerous ideas and suggestions for engaging and gameful ways to develop and design the *MyStrengths* app. The final phase saw the output from all the earlier phases come together. Through multiple increasingly complete iterations of user evaluations testing and developing, the final prototype of the *MyStrengths* app was created.

**Conclusions:**

Although research supports the use of strengths-focused mHealth tools to support people living with chronic illnesses, there is little guidance as to how these tools and their content should be designed. Through all activities, we found great support among participating users for strengths-focused apps, and we can consider such apps to be both appropriate and valuable. This paper illustrates how combining a range of user-, researcher-, literature-, and designer-based methods can contribute to creating mHealth tools to support people with chronic illnesses to find and use more of their own personal strengths.

## Introduction

### Background

The number of people living with chronic illnesses is continually increasing [1,2]. Between 2010 and 2020, the World Health Organization has predicted the number of persons with chronic conditions to rise by 15% [3]. For instance, in the United States, 6 in 10 people have one chronic disease, and as many as 4 in 10 have 2 or more chronic diseases [4]. Living with chronic illnesses is a highly demanding task, and many of the challenges faced are shared across conditions and demographics. For instance, chronically ill people can be required to cope with symptoms, disabilities, medication regiments, lifestyle changes, or emotional consequences such as depression and fear [1,5,6]. Managing these aspects of life with chronic illnesses is often described as self-management [7,8]. Traditionally, self-management interventions in health care have focused on patients’ actual or potential health problems, risks, and deficits [7,9]. This is usually done by providing support to solve, alleviate, or prevent such problems through information, skill training, and teaching coping techniques. However, evidence from psychology and biobehavioral sciences points to the traditional health care approach, that is, focusing on people’s problems and deficits, as not optimal to aid people in reaching their best health potentials [10,11]. Increasingly, self-management interventions include more balanced approaches that not only assess a person’s deficiencies (eg, symptoms, problems, and needs) but also integrate positive personal resources and values. They are, in essence, providing a more holistic approach to health and well-being [8,12,13].

As the group of people living with chronic illnesses grows, a readily available mHealth tool that is not disease specific but instead aims at supporting its users overcome commonly shared challenges could be of great use. One particularly promising approach to provide support anchored in an individual’s positive personal resources and values is to help them in recognizing and using more of their personal strengths [10,14]. In this paper, we describe the process of creating such a tool, the *MyStrengths* app.

### Personal Strengths

The concept of personal strengths has its foundation in positive psychology [10,15] and has been defined as “traits/capabilities that are personally fulfilling, do not diminish others, ubiquitous, and valued across cultures, and aligned with numerous positive outcomes for oneself and others” [14]. Simply put, this means emphasizing what is possible, valuable, and doable, as opposed to focusing on deficits and problems [16,17]. A focus on people’s own strengths has been shown to contribute positively to better moods and happiness [10] as well as increased general health and well-being [14,18,19]. Focusing on health care, Sturgeon and Zautra [20] reported how people with chronic pain use traits such as positive emotions, optimism, and social engagement to maintain a good life. Similarly, Rotegård et al [21] found cancer patients to employ strengths items such as will power and trust in health care providers to meet their daily challenges. In a study on adults with major depressive disorder, Cheavens et al [22] found better outcomes from personalizing treatment to focus on the patients’ strengths rather than on their deficits and problems. In a study among people with one or more chronic conditions, positive emotions have been connected to increasing patient activation [23]. In addition to the strengths reported in the studies cited earlier, a multitude of different strengths used to overcome challenges and live a good life has been identified by participants in studies on strengths among people living with chronic illness in general: having a positive outlook on life; being persistent; being kind and caring; having courage; having support from family, friends, peers, and health care providers; and having constructive self-management strategies [16,24,25].

In sum, being aware of and mobilizing one’s personal strengths can lead to a wide range of positive effects on health and well-being both for people in the general population as well as people living with chronic illnesses.

### mHealth and Design for Engagement

In addition to the increasing ubiquity of smartphones and personal computers, both electronic health (eHealth) and mobile health (mHealth) interventions have been developed to support a wide range of health-related goals, for instance, medication adherence [26], symptom monitoring [27], support of smoking cessation [28], managing rheumatic and musculoskeletal diseases [29], and stress management [30]. However, to our knowledge, no tools have been designed focusing on identifying and mobilizing people’s personal strengths in support of people living with chronic illnesses.

Although mHealth tools or services show great promise for supporting people with chronic illnesses [31], their success is often contingent on them being used as intended [32,33]. However, focusing on use alone is not necessarily enough, and as Kelders [33] argues, one should also take into consideration users’ sense of involvement with and enjoyment of the mHealth tools. Together, these 3 aspects make up what can be referred to as engagement [34,35]. To increase users’ engagement with tools or services, the use of design approaches known from the world of games, typically called gamification or gameful designs, is increasingly popular [36-39]. There are several definitions of gameful designs, and in this project, we define it similar to the one presented by Huotari and Hamari [40]: using design approaches and implementations from the world of games (in our otherwise nongame tools) to add a sense of playfulness and increase users’ enjoyment and engagement. To date, gameful eHealth and mHealth tools have been designed for a variety of purposes, such as mental health [41], smoking cessation [42], and promoting physical activity among patients with rheumatoid arthritis [43].

Concerning the efficacy of gameful designs, Johnson et al [36] reviewed gameful designs for health and well-being. They found a positive influence of gameful designs for tools aimed at increasing physical activity and fitness, nutrition, health care utilization, medication misuse, blood glucose monitoring, and patient empowerment. Importantly, the authors also found gameful designed tools to have positive effects on personal growth, well-being, flourishing, stress, and anxiety. Thus, as they have the potential to positively affect not only behavior but also users’ well-being through positive and engaging experiences, gamefully designing tools or services should be particularly promising within the field of health [36,38].

Looking at specific gameful design techniques and implementations, the use of *points, badges, and leaderboards*, often referred to together as the PDB triad, is the most used approach among gameful interventions [36,37]. Such approaches have been critiqued for simplifying the nature of human motivation by assuming that humans primarily are motivated by achieving increasingly more or by merely leading or winning over others [44,45]. Gameful design approaches, such as chasing rewards, have also been reported as unfitting for mindfulness and well-being interventions [46]. It should be noted, however, that gamified well-being interventions have also been designed successfully using the aforementioned approaches [38].

Thus, when designing a tool that centers around self-awareness and reflection, it appears crucial to create engagement in ways that are aligned with the users’ real interests and values. This can, for instance, be done by having the app represent *a* *better self* of its users with avatars and creating an involving and interesting narrative, rather than only external factors such as points and leaderboards [45,47]. Although research points to the positive effects of gameful designs, no evidence-based framework for designing eHealth or mHealth interventions gamefully currently exists [48,49]. Furthermore, what the *active ingredients* of successful gameful designs are is mostly unknown [36].

### Participatory Design Processes

Users’ participation in the design process is a productive way of ensuring that gameful designs are experienced as appropriate as well as meaningful to the end users [48,50-52]. Contributing to the literature on participatory eHealth development, this project takes a *participatory design* approach [53] to investigate ways of designing and creating the *MyStrengths* app.

Participatory design is guided by the fundamental ethical stance that the end users, whose future may be affected by the design, should have a say in the process. As such, participatory design processes do not merely include users as passive informers and evaluators but seek to include these as co-designers throughout the design process [54,55]. As such, participatory design is not merely concerned with collecting users’ needs, ideas, or preferences but also with enabling meaningful participation. Examples include using techniques such as design games [56], role-playing [57], or future workshops [58]. In eHealth and mHealth, participatory approaches have, for instance, been involved in the design of apps to support teenagers with chronic illnesses in the transition from pediatric to regular care [59], to support young children living with cancer [60], and to facilitate stress management for cancer survivors [30].

### Aims

As presented, the number of people living with one or more chronic illnesses is increasing. The rising ubiquity of smartphones affords mHealth research and designers to create support tools that can easily reach a large number of users without relying on their physical access to health care services and personnel. As personal strengths are common to us all, a tool supporting people living with chronic illnesses to find and use more of their own should thus be of great benefit. To our knowledge, neither the development nor the evaluation of any such tool has been published before.

Addressing this, our main goal was to present the activities, discussions, and decisions undertaken during designing the *MyStrengths* app. Our secondary goal addresses the lack of guidelines for creating both gameful designs and strengths features or activities for people living with chronic illnesses. Through this project, we explore and evaluate ways to integrate the focus of people’s personal strengths into mHealth tools and how to make such tools more engaging through gameful and engaging designs in ways that are suitable for, respectful to, and appreciated by people living with chronic illnesses.

## Methods

### Overall Project Design

This paper presents activities from the project “The Power of Personal Strengths—using gamification to support patients in chronic illness management.” The project was conducted at the Department of Digital Health Research at Oslo University Hospital, Norway, between 2016 and 2019, and funded by the Research Council of Norway (grant #248026).

To achieve its goal of creating the *MyStrengths* app, the project has a comprehensive, iterative, and participatory approach to combine patients’ preferences and requirements with knowledge and expertise from the fields of self-management, positive psychology and well-being, and mHealth design. The overall design process follows the 4 stages presented in the Double Diamond design process [61]: discovering, defining, developing, and delivering. The 2 first and the 2 latter stages make up a diamond of diverging and converging activities. Divergence refers to activities where designers (and co-designers) go broadly out to discover new opportunities, solutions, and ideas for their design. Conversely, the converging activities are focused on narrowing down, concretizing, and creating based on the former phase [62].

### Participants

The main project team consisted of 5 researchers (with backgrounds in nursing, public health, behavior change, informatics and design, and mHealth development), 2 editorial content producers, and a patient representative, to ensure that the user perspective was maintained. Although the project, outside of one of the activities, does not directly include health care workers, it should be noted that 4 of the 8 participants in the group had long clinical working experiences. The team met weekly and planned and coordinated the work throughout the entire project period.

In addition to the main project team, the project employed 3 main types of participants during the activities:

1. People in the user group, living with chronic illnesses. These people were recruited through collaborating institutions, such as learning and mastery centers at local hospitals or patient organizations, or from the team’s professional network. Recruited in the same manner, patient representatives and caregivers as well as designers and researchers took part during the first phase.
2. Designers and developers, external and from the in-house information technology (IT) system development group.
3. Researchers who are part of the project’s international advisory group.

### Ethical Considerations

The project was planned and conducted in adherence to the principles of the Helsinki Declaration [63] and approved by the Privacy Protection and Data Security Committee at Oslo University Hospital. All participants, or their legal guardians, signed informed consent before taking part.

## Results

### Design Activities and Results

The following section presents the iterative activities and the outcomes from the development process. As each phase builds on the earlier phase, the activities and results are presented grouped into the 4 phases of the design process:

1. In the discovering phase, users and other stakeholders took part in a full-day workshop, working toward identifying and ideating ideas for how a strengths-focused mHealth tool could work and be designed.
2. In the defining phase, inputs from the earlier phase as well as from literature and previous projects and experiences were discussed, and decisions on content and priorities were made.
3. The developing phase focused on the user experience of the tool.
4. In the delivering phase, all information and input gathered were put together, and a working high-fidelity prototype of the *MyStrengths* tool was developed.

**Figure 1:**
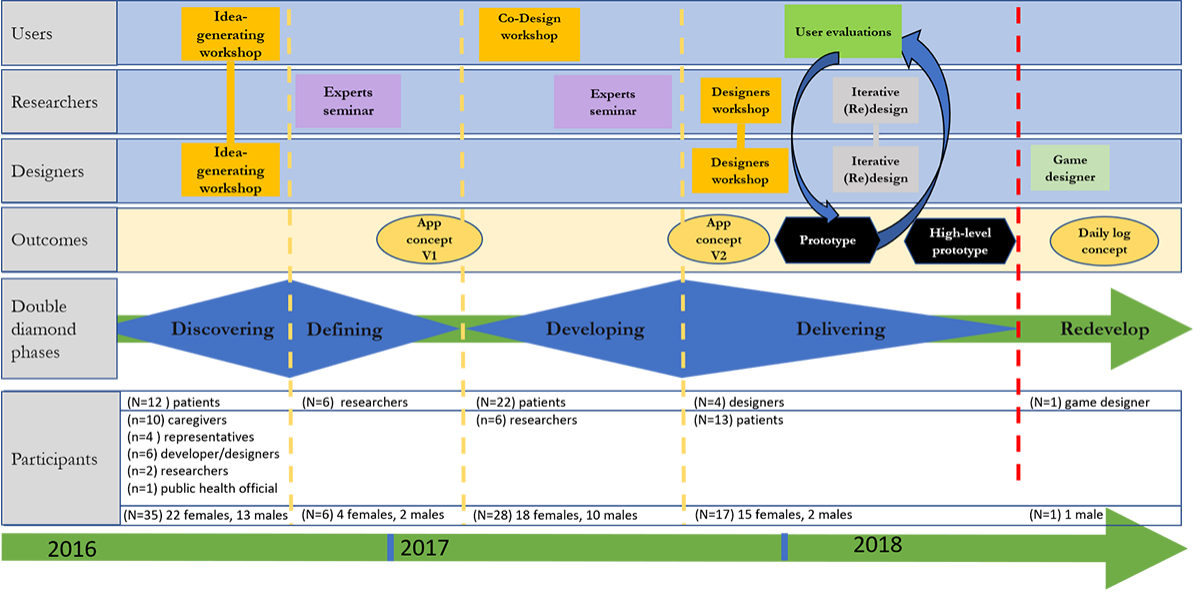
The project’s phases, activities, and participants.

A visual overview of the project’s phases, activities, and participants is presented in Figure 1.

#### Phase 1: Discovering

The first phase of development explored, through an idea-generating workshop, how a mHealth tool could be created to support people living with chronic illness through discovering and using their own strengths.

##### Methods and Activities

###### Idea-Generating Workshop

**Figure 2:**
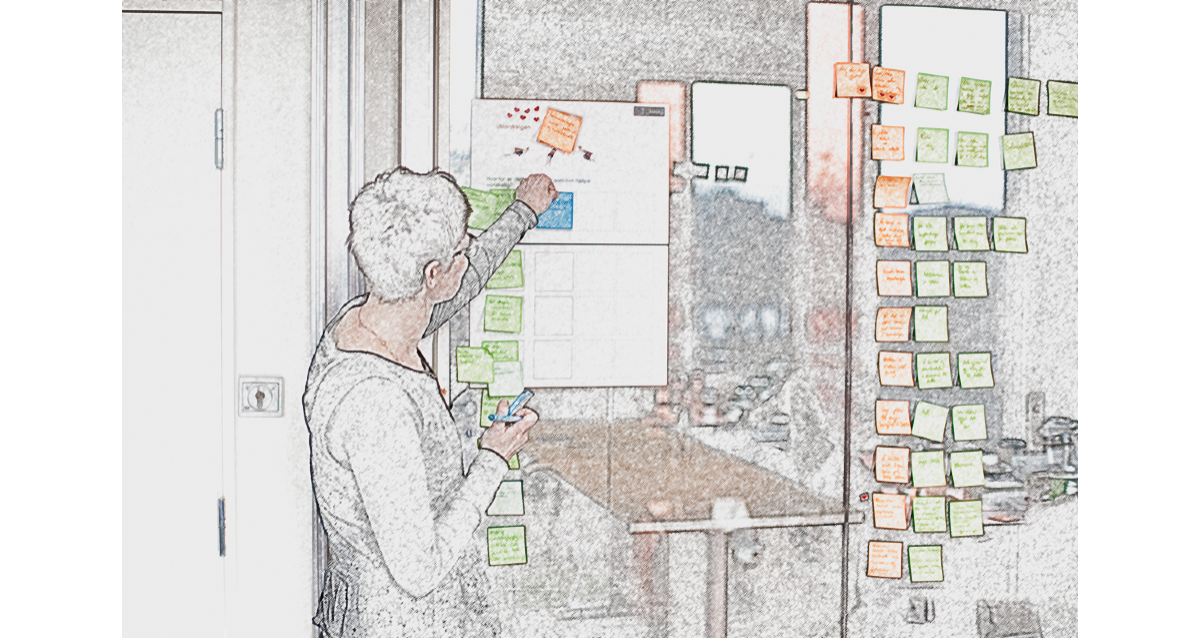
Activity from Idea Generation workshop.

To gather ideas and input from relevant stakeholders, a day-long idea-generating workshop was hosted, where patients, relatives, representatives from patient organizations, health care personnel and researchers, and designers and developers participated (n=35). The design of the workshop was inspired by Future Workshops, a common idea-generation activity within participatory design [57], and additionally adjusted with a positive and forward-looking approach based on principles from *appreciative inquiry* [64]. The participants worked in 6 groups hosted by members of the project team, and the first part of the workshop consisted of group activities focused on identifying and presenting each person’s personal strengths, naming typical challenges experienced by people living with chronic illnesses, and what strengths people could use to overcome these. An illustration of this activity is shown in Figure 2. The latter part of the workshop had the same groups create concepts for an IT tool that could help them use their own strengths to overcome their daily challenges in a new and innovative manner.

In addition to the briefly described findings presented here, a detailed description of workshop methods and results are described in a separate publication [65]. Data from the workshops were qualitatively analyzed by the first and fourth authors of that publication using thematic analysis [66].

##### Results

###### App Concepts—Strengths Focus

Each of the six participant groups created concepts for different eHealth tools that could support them in overcoming the selected challenges.

**Figure 3:**
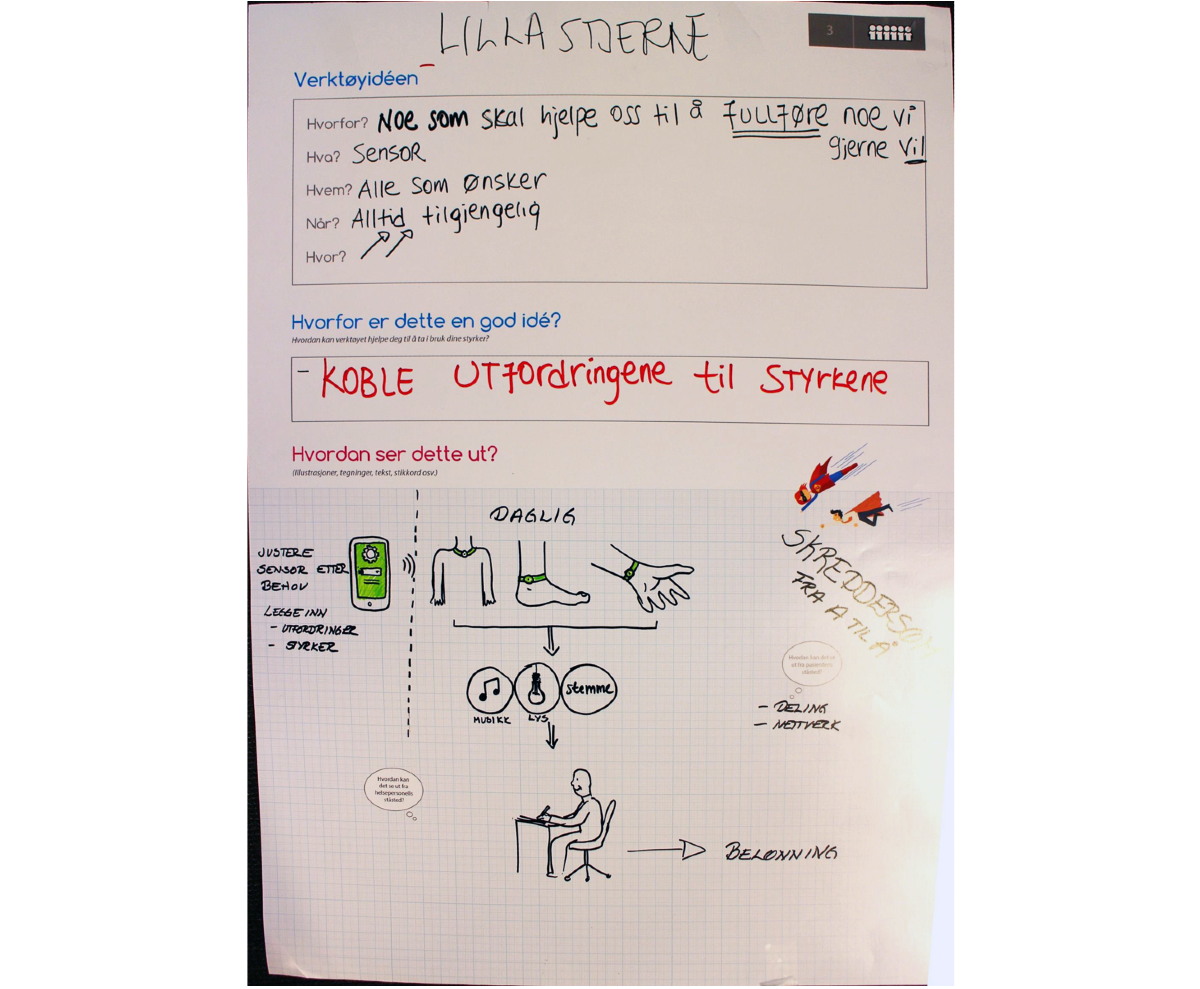
Example of User-Concept.

For example, Figure 3 shows the concept created by one of the participant groups. The suggestion is a personalized app that is designed to help the user identify her strengths and use them to complete challenges and tasks in everyday life. It could, for example, integrate sensors to interpret when a person is stressed and then prompt her with some of her strengths to help and motivate her to complete initiated tasks. The group participants also suggested more specific ideas for the strengths focus in mHealth tools, for instance, (1) having friends in the app suggest strengths the user has; (2) that the app provides the user reminders of her strengths; (3) based on previous input in the app, providing tips for what strengths the user can use to overcome new challenges; and (4) that the tool could offer exercises and tasks that would either build on or provide opportunities for the users to mobilize and use their own strengths.

Table 1 presents the 6 groups and their mHealth tool suggestions. We planned to distribute the participants evenly between the groups based on gender and backgrounds, although some changes were outside our control. Although the groups vary in composition and only some of the ideas explicitly focused on mobilizing the personal strengths among people living with chronic illnesses, a positive and strengths-focused foundation exists in all the app ideas.

**Table 1:** Six proposed mobile health or electronic health tools from the idea-generating workshop (N=35, 13 males).

Group number (participants)		App idea	Challenges	Description
**1 (n=5, 2 males)**		Strengths treasure chest	Finding balance in life	A treasure chest app where a person can store his or her strengths, those written by himself or herself as well as strengths added by others (eg, friends and family)
	Patient (n=1)			
	Health care provider (n=1)			
	Public health official (n=1)			
	IT^a^ developer (n=1)			
	Researcher (n=1)			
**2 (n=8, 3 males)**		Cheering squad app	Mastering various aspects of life	An app where a person can invite people to join his or her own cheering squad
	Patient (n=4)			
	Health care provider (n=3)			
	IT developer (n=1)			
**3 (n=6, 3 males)**		User-controlled personalized hospital	Challenging to be a young adult in a hospital	An app for a person transitioning from a pediatric ward to a unit for adults in a hospital
	Patient (n=2)			
	Health care provider (n=2)			
	Researcher (n=1)			
	IT developer (n=1)			
**4 (n=7, 1 male)**		Empathy simulator	Communication among relatives and the health care system	Virtual reality four-dimensional glasses that simulate experiences from different parties present in a consultation setting (eg, patient, caregiver, or a family member)
	Patient (n=1)			
	Patient organization			
	Representative (n=2)			
	Health care provider (n=3)			
	IT developer (n=1)			
**5 (n=6, 2 males)**		Prioritizing app	Prioritize among important things	An app to help a person to make choices based on earlier knowledge and experiences
	Patient (n=3)			
	Health care provider (n=1)			
	Patient organization			
	Representative (n=1)			
	Designer (n=1)			
**6 (n=3, 1 male)**		Task completer app	Finishing projects	An app that helps a person identify his or her strengths and use them to complete tasks in everyday life
	Patient (n=1)			
	Health care provider (n=1)			
	Designer (n=1)			

^a^IT: information technology.

###### User Requirements and Functionality Ideas

The analysis of the workshops revealed 4 main themes of functionality requirements for the strengths-focused self-management tool:

1. Social support, that the tool should include support for social support and interaction, for example, by providing the possibility to chat with peers or role models, or one-directional messaging that friends can send to the user to cheer him or her up but that does not require the person to respond.
2. Supporting patient-health care providers’ collaboration, for example, by supporting communication with providers, preparation for consultations, or easy sharing of information. One group suggested giving the user the possibility to use the app to give providers better insights into their feelings and values and using this information so that the treatment could be adjusted to the best fit these.
3. Awareness and reflection, allowing users to develop awareness and reflection about oneself and one’s current situation. This could, for instance, be done by adding to and adapting the app to fit the users’ values and situation or by helping the users to be more aware of, and use, their own strengths.
4. Supporting the users with coping strategies, by, for example, helping them to prioritize and make choices between different activities and goals that they wish to do or accomplish and by providing an overview of activities, goals, and choices done in the past.

###### Users’ Preferences and Needs for Design and User Experience

The workshop also revealed a wide range of user requirements for the design of the strengths tool itself. The most prevalent requirement is that the design should have an overall positive focus and approach. For instance, it was suggested that all feedback be given in a supportive and positive manner or using *pleasant* and engaging metaphors such as treasure chests, islands in an ocean, or avatars. The participants also considered it important to be able to customize and adapt the tool to their own needs and preferences. This could include adjusting the tool content depending on the user’s diagnosis or health situation, their level of experience, and simply being able to turn functionalities on or off.

#### Phase 2: Defining

To further inform the project, the second phase mainly consisted of us performing a literature search on strengths-focused interventions. These findings, as well as the outcomes from the first phase, were then discussed and evaluated in a research seminar.

##### Methods and Activities

###### Literature Review

To further explore the topic of strengths-focused management and support of individuals with chronic illnesses, we conducted a literature search using terms that employ positive approaches (eg, positive psychology interventions and mindfulness interventions). Although this review was intended to be published, the resource situation and practical changes on the project would regrettably thwart this. The findings are nonetheless outcomes of the project and are, therefore, presented in this paper.

###### Research Seminar

We hosted a 3-day research seminar with the main project team and 6 experienced national and international researchers from the field of behavior change, psychology, eHealth, service design, and participatory care. The primary goal of this research seminar was to combine the results from the previous phase with knowledge, evidence, and previous experiences to decide on a set of core features for the *MyStrengths* tool. Although the user representative was the only user taking part in the seminar, we took care to ensure that the opinions, needs, and ideas voiced by users were present and given equal weight during discussions and decisions. A secondary goal of the research seminar was to discuss the findings from the review of previous literature and how these findings might be used beneficially in the project.

The activities in the research seminar took many forms, including presentations, brainstorming, discussions, and versions of the activities from the idea-generating workshop held in the previous phase. The seminar was audio recorded, and a detailed summary and a key item take away form was created using these recordings as well as participants’ notes, drawings, and photos.

##### Results

###### Review

From an initial search response of 6742 records, 7 publications on 6 different interventions were identified and included in the analysis. Three of these were delivered as in-person or face-to-face interventions [67-70], 2 interventions were delivered through web-based channels [71,72], and 1 intervention consisted of both offline and online intervention delivery modes, one per intervention group [73]. In all studies, personal strengths were implemented as one part of the intervention, and none of the studies focused solely on personal strengths. In all, a lack of consistency in the literature was found, and personal strengths were scarcely covered. This is also a common finding in more extensive reviews on behavior change techniques [74].

Of the more promising findings, the study by Nikrahan et al [67] identified a positive effect on hope and happiness at a 15-week follow-up of the participants. Participants were randomly assigned to 1 of the 3 positive intervention groups or a wait-list control group, and participants in the intervention groups received a 6-week in-person group training program. Two of the intervention groups included strengths activities. In the first group, participants were asked to identify a *signature strength* from a list of 24 personal strengths, use a signature strength in daily activities and identify strengths in their partners and children, and use a signature strength in a way that furthers a cause larger than oneself. In the second group, participants in parts of the intervention focused on positive personality traits to overcome fears about others’ opinions; accept themselves; and initiate contact with people they would like to meet to foster authenticity, self-esteem, and extroversion. In another study by Cerezo et al [69], the experimental group received weekly face-to-face sessions aiming to provide positive psychology-related coping strategies and enhance their psychological strengths. From this, the authors found positive between-group effects on positive emotions postintervention when comparing the experimental group with a control group.

In addition to the activities described earlier, strengths activities employed in all the studies identified ranged from questionnaires asking participants to select strength(s) items that most apply for them or naming strengths directly to exercises where they reflect on how they have used their strengths recently or aim to use a signature strength in a daily activity. Although such exercises are in line with common approaches for identifying personal strengths [14], none of the included studies reported on adaptations of strengths exercises and activities to the specific target groups.

A brief description of the 6 identified interventions’ designs and findings is presented in Multimedia Appendix 1 [67-73]. As these interventions typically consist of multiple modules stemming from separate theories or approaches, it was not possible to conclude on either specific effects or mechanisms through which the interventions affected well-being. However, other studies have previously identified goal setting as a potential promising factor. More specifically, Linley et al [75] have shown strengths use to be associated with goal progress, which, in turn, was related to psychological need fulfillment and enhanced well-being in the general population, thus providing an example of how positive interventions could be combined with self-management interventions to support patients in better management of chronic illnesses.

###### Research Seminar Discussions and Project Development

When discussing review findings and further plans, we thoroughly discussed the overall approach of the *MyStrengths* tool and how it should be designed for the best possible effects. On the basis of the lack of evidence for specific mechanisms for increasing people’s use of strengths, a general 3-step approach was suggested: (1) create awareness of people’s strength; (2) help users to reflect on these; and (3) support users in using the strengths by, for instance, setting small and achievable goals. This was also along the lines of 2 of the identified publications [72,73], which also integrated personal strengths in the same module as personal goals. Concluding this phase, the outcomes from the idea workshop in the previous phase were presented, discussed, and sorted into main categories of possible features for the *MyStrengths* tool. These features are shown and briefly described in Textbox 1.

**Suggested *MyStrengths* tool features.**

**My strengths**

Assessment and overview of the user's own personal strengths.

**My goals**

Larger goals the users want to achieve and can use his or her strengths to reach.

**My small experiments**

Small goals or activities that can serve as building blocks on the road to achieving the user's larger goals.

**Exercises**

Activities or tasks the user can do to use his or her strengths more. These can be created by the user or be preprogrammed within the app.

**My experiences**

A logbook that would allow the user to write down and reflect on activities or situations in relation to how they did or did not use their strengths. These reflections could then be accessed at a later stage and form part of the users planning or reflection on new activities, thus help them in using their strengths more productively.

**Information**

Content that explains as well as expands on the concept of strengths and its scientific background. This section could also provide specific information for the users, for instance, based on specific illnesses or a geographic area.

**Social support**

A variety of social features that could allow the user to communicate, support, or get support from other users, for instance, by sharing experiences or by supporting each other in reaching goals.

**Timeline**

A section of the app that would gather all input information in one place and present it in a visual and nice fashion.

**About me**

A profile page the users can set up to describe themselves, their values, and what is important to them.

**Settings**

General settings for the app.

#### Phase 3: Developing

Having created a set of features or functionalities of the *MyStrengths* tools, the goal for the third phase was to further develop and hone ideas and concepts for how to best design and implement features in an engaging and motivating way for users. This phase consisted of 2 main activities: a series of co-design workshops with users and another research seminar with experts in the field.

##### Methods and Activities

###### Co-Design Workshops

With the suggested features of the *MyStrengths* tool as a starting point, design challenges and low-fidelity mock-ups were created and used in a series of co-design workshops. In total, 2 workshops were conducted with each of the 3 different participant groups, from 2 educational centers and a youth council from hospitals in Norway.

Each of the workshops used participatory design methods, including design games, prototyping, and scenario making [57,76,77]. The first workshop focused on the design of the tool in a gameful and engaging way, and the second workshop built on the former and focused on the users’ discovery and use of their own personal strengths into such tools. In addition to providing new ideas for the design and features of the *MyStrengths* tool, the workshops also allowed the participants to give feedback on the ideas and features that had been mocked up as part of the design activities. The detailed descriptions of the methods, procedures, and outcomes of these workshops have previously been presented in a separate publication [78].

###### Research Seminar

Next, a second 3-day research seminar was hosted with the project team and the same research experts from the fields of behavior change, positive psychology, eHealth, and participatory care taking part. As with the earlier seminar, the user representative was the sole participant from this group. Still, we also took great care to communicate and represent the perspectives and inputs stemming from user participants through all workshop activities. The seminar’s goal was to discuss and gain feedback on the undertaken activities and their outcomes to confirm these and to prioritize features and functionalities for the tool. A third focus of the seminar was to inform and guide the team in the design and content development of the strengths exercises to be included. As with the seminar in phase 2, a range of activities such as presentations, brainstorming, and discussions were employed. To further the participants' understanding and appreciation of the user ideas from the earlier activities, we also used tasks from the co-design workshop, with participants splitting into smaller groups. The research seminar was audio recorded, and the recordings as well as participants’ notes, photographs taken, and drawings were used to create a detailed summary and a listing of key decisions, discussions, and takeaway points.

##### Results

###### Co-Design Workshops

In total, 22 people with various chronic illnesses, in 3 locations, participated in 2 consecutive co-design workshops (see Table 2 for participants’ age and gender). The workshops revealed further requirements from the user group in the form of numerous suggestions for how game-like features and elements could be designed to create a strength-based eHealth and mHealth tool.

**Table 2:** Participants in co-design workshops.

Group number	Participants, n	Age (years), range
Group 1	7 (2 males)	17-21
Group 2	7 (2 males)	21-58
Group 3	8 (3 males)	27-64
Total	22 (7 males)	17-64

In the workshops, the participants created posters and wireframes with their ideas for using gameful designs in strengths-focused mHealth apps. During the second workshop, they also rearranged and commented on simple mock-ups of the app created based on output from the first workshop. As with the idea-generating workshop in phase 1 (discovery), the importance of keeping the user experience positive and supporting emerged as one of the most important requirements. Typical and proverbial gameful design elements such as points, competitions, or trophies [37] were also often suggested. The participants also voiced several concerns, for instance, the need for the new tool to be experienced as motivating, yet not addicting, by overly focusing on scoring points and winning.

The design workshops also yielded feedback and input into using mHealth to support people in using their strengths more. For instance, one group suggested a strengths assessment system in which the tool narrows down one’s strengths by asking a series of questions and then suggesting strengths one might have based on the answers.

**Figure 4:**
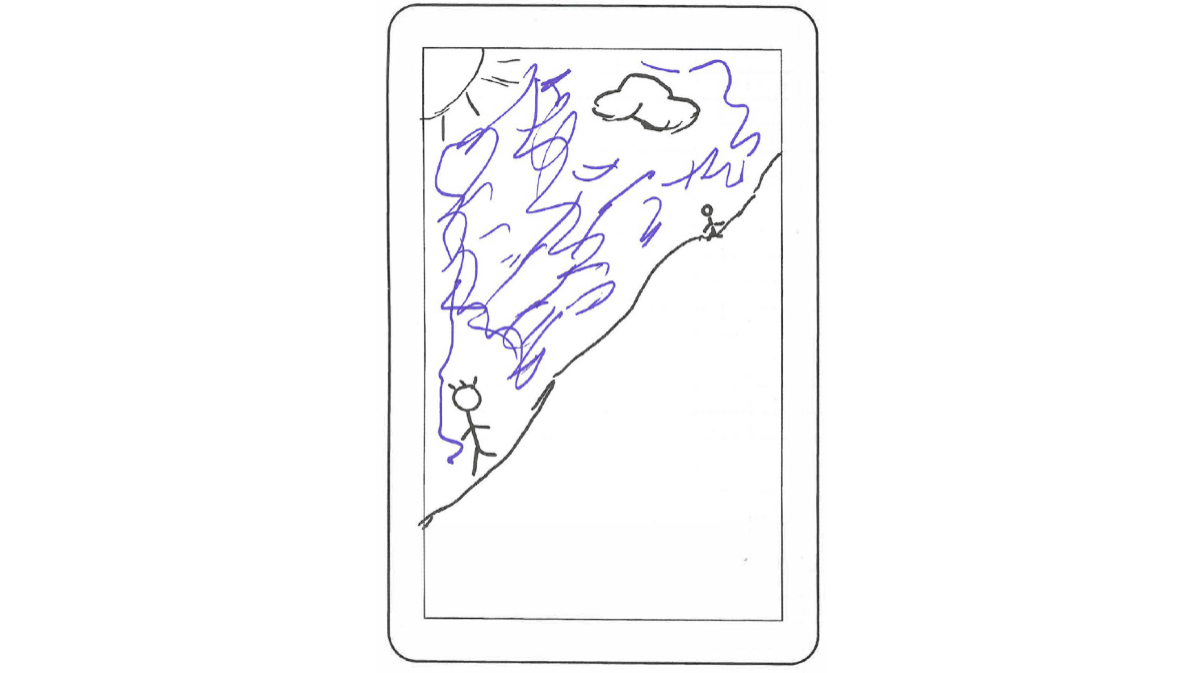
User sketch of idea.

Some of the groups created complete concepts for self-management apps. For instance, Figure 4 presents a sketch of a progress tracker in the app, visualized as the user and a friend competing to reach the top of a mountain. Other concepts further provided the user with opportunities to collaborate and share experiences or activities with friends as well as the option to set increasingly hard goals and targets. In terms of collaboration, it was also suggested that one could send anonymized small predefined texts or icons to other users as an uncomplicated way of creating social features while maintaining user privacy.

##### Research Seminar

###### Features and Functionalities

In the research seminar, the proposed list of features and functionalities was thoroughly discussed in light of previous experiences and literature as well as with regard to the outcomes from the co-design workshops. One of the chief decisions made was to focus the remaining work on functionalities directly related to the user's strengths and the use of these, rather than features providing information or social interaction. Providing information was discussed and dropped as users, in general, were considered to already have considerable access to relevant information fitting their specific needs. As such, creating relevant content surpassing what is already available to the individual users was considered to be very time and resource intensive. This has also been reported by participants in the design workshops and is additionally supported in the literature [79].

The social features were cut primarily because of regulations from the privacy protection committee at our institution. To log in to patient-facing services that have social and/or sharing capabilities, even if this information is anonymized, these regulations mandate the use of a level 4 two-factor authentication system. These are, in Norway, available from a limited number of approved providers and are typically used to log in to banks and public services. Both the researcher’s experiences and direct statements from participants in our various workshops pointed to this being overly cumbersome and would lead to the app not being used much. As such, designing ways to create social interaction between users was deemed challenging and resource intensive, and we were forced to drop these capabilities.

As a way of supporting users to easily use their strengths more, it was suggested to link specific strengths to goals and then have small, more easily achievable subgoals. It was proposed that this connection could be made by starting with a goal and then finding a strength or simply by starting with a strength the users want to use. The overall goal of this is to create a list of the personal strengths the user has. The app should also present the user with suggestions for how they could use their strengths in what the project team described as strengths exercises, in case the participants did not come up with activities themselves. The tool could then provide the user with feedback and positive reinforcement on the goals accomplished. An underlying goal should be to change the user’s habits in small steps, as typically done in behavior change interventions [80], and help them gradually use their strengths more. To further contribute to users' well-being, it was also suggested that the app also should feature gratitude exercises, such as the *three good things* exercise [10,14].

###### Design

In terms of design, the second research seminar resulted in the overall idea of designing for gameful and enjoyable interactions that could help users learn for their own experiences and be more active in their self-management. During discussions, particular focus was placed on the users' first interaction with the tool and how this should be designed to simultaneously explain the rationale behind a strengths focus, introduce the app, and motivate the users to start exploring their strengths. For example, it was concluded that using avatars could be a positive source for motivation, as it could provide users with social motivations and support and, in some cases, allow the users to visualize a better self. Some suggestions included designing avatars to fulfill the role of either a guide or a companion or a narrator to the app (for instance, a friendly avatar to be one’s climbing companion) or as a virtual representation of the users (designed by the users themselves during the initial use of the tool). Another way proposed to provide relatedness with the app could be using videos (animated or real life) that could present the concept and rationale of strengths or compelling user stories.

The use of different metaphors in the app was proposed and discussed. Among other ideas, an expansion of the user-generated *climbing mountains* concept from the co-design workshop was suggested. Here, the user would still have climbing mountains as a goal, and the way to get to the top would contain several stops and base camps where the user could reflect and take stock of the tools (strengths) they are using for the expedition. Other ideas were to theme the app as a journey of discovery. Still, after discussion, a decision was made that a potential metaphorical theme or approach for the tool should be culturally neutral and not focus on metaphors that, for instance, are well known by some populations but can be unfamiliar to others. More traditional game features, such as points and unlockable content, were also suggested. However, a consensus opinion was that these should only be implemented if they could provide further value to the tool as a whole, as opposed to merely adding points for the sake of gamifying the tool.

At the end of this phase, the feature list for the *MyStrengths* tool was updated by removing, adding, and reorganizing features into a more detailed list. Specific ideas and features suggested by users and researchers or identified in the literature were added and annotated with its source. Although the overall categories were supported and suggested from users, researchers, and literature, the specific implementations of these often differed. For instance, at the end of an exercise, users suggested performing a reflection task on the difficulty of doing the task, whereas the researchers suggested performing a reflection task concerning how you had used your strengths to complete the task. When completed, the list (presented in Textbox 2) covered 4 main sets of features.

**Intervention feature overview list following phase 3.**

**My strengths**

Assessment and overview of the user's strengths using a predefined list of strengths

**Strengths exercises**

Examples of exercises the user can try out on one or more of his or her specific strengths to try out to apply these in his or her daily life in a new way

**Strengths experiments**

Small activities that can serve as building blocks on the road to achieving the user's goals. Here, the user would first outline a goal and then plan out several small activities, or experiments, that could help toward reaching it

**Daily log**

Section of the app with 2 separate features. First, it allows the user to rate how the day has been and write down the *three good things* exercise. Second, it collects and visualizes the user’s inputs and activities throughout the app and finally provides a summary for each day in an easily scrollable interface

#### Phase 4: Delivering

With a core set of features, the fourth phase of the project consisted of the final design and the technical development of the *MyStrengths* app through workshops with designers, development with our IT department, and iterative evaluations from users.

##### Methods and Activities

###### Designer Workshop

With the 4 primary features for the app (ie, strengths assessment, strengths exercises, strengths experiments, and daily log) as a basis, a 2-day design workshop was held to condense the gathered inputs and create a central concept for the tool. In addition to the core members of the project team, our in-house designer and one developer as well as 4 external designers experienced in designing for health and behavior change participated. For these workshops, inspiration was drawn from how *game jams* are organized [81]. Starting with a thorough description of the activities undertaken thus far, the participants worked in smaller groups. During the second day, a concept emerged, and the latter part of the workshop was spent on collectively elaborating and embellishing this concept.

###### Strengths and Strengths Exercises

With previous research on personal strengths at our research center as a basis [16,21], a list of 40 strengths to be included in the assessment part of the *MyStrengths* app was created. Connected to each of these strengths, we then created matching strengths exercises. These exercises are activities that the user can perform to employ a specific strength. For instance, if one person has kindness as a strength, the app could suggest for him or her to do something kind to a neighbor today. Most of these exercises were based directly on strengths exercises found in academic and popular literature [10,14,82-84] or related to items reported by participating patients in earlier related studies [16]. The wording and content of these exercises were iteratively refined during the user evaluations of the app.

###### Iterative Development—User Evaluation

Building on the outcomes from the workshop with the designers, the research team worked closely with the in-house designers and developers to sketch out and create mock-ups of the specific features of the app.

Over 4 iterations, with more and more of the features added, the app was evaluated by people living with chronic illnesses. On 3 occasions, colleagues at our center experienced in eHealth and mHealth development, and new to the tool, also evaluated the app using the same setup and methods as the evaluators from the user group. On the basis of feedback from the evaluations, adjustments were discussed and made by the research and development teams.

**Figure 5:**
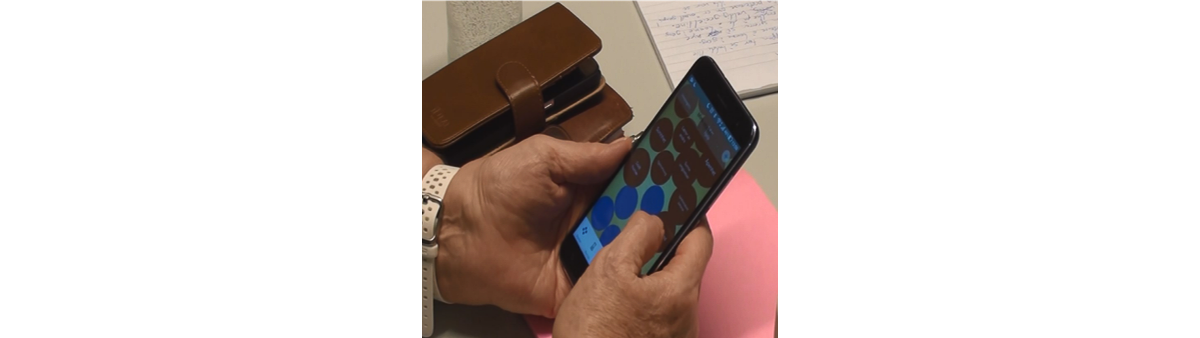
User evaluating the app (capture from video).

All user evaluations were audio and video recorded (see Figure 5 for an example of the video frame). The first author (SJ) hosted the evaluations, whereas a member of the project team observed, supported the host when needed, and took notes. During evaluations, participants were not given instructions but were told to navigate through the app freely and describe their impressions and actions by thinking aloud [85]. Having gone through all the features available, the user and the project members openly discussed the experience and any thoughts or ideas the user might have. Both the facilitator and the observer wrote detailed notes. The first author rewatched the recorded evaluations and, drawing on these and the notes, made detailed reports from each evaluation. Individual reports were combined and presented to the research team and developers. This group then discussed the findings and ideas from the evaluations and then decided on what changes to make to both existing and planned functionalities and design.

##### Results

###### Designer Workshop and Iterative Development

**Figure 6:**
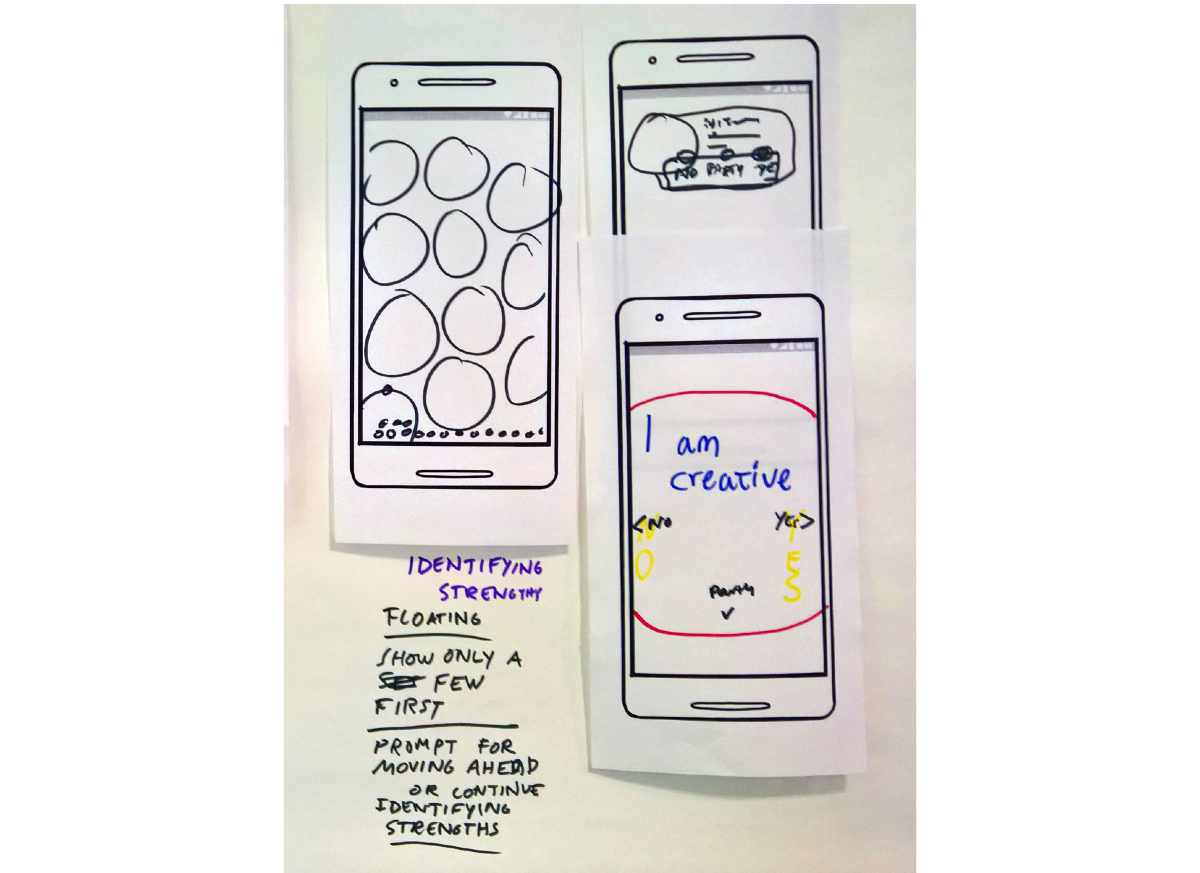
Home Screen Sketch (left).

During the designers’ workshop, on advice from researchers and experts, it was decided that concrete metaphors should be avoided. Following this, we decided on a concept with spheres visualizing the users’ strengths floating on the home screen. See Figure 6 for an early sketch of the home screen. By putting the users' own strengths front and center, simply opening the app could serve as a positive reminder of all the strengths the user has. When starting the app for the first time, a single sphere would float up to the top of the screen. The users can then click on and rate the strength as having, partially having, and not having. The app includes 40 strengths, and based on the ratings given, the spheres have different colors. To emphasize the strengths the user has, the ones rated as having would float on top. Under these, the other spheres of the partially having would float, and at the bottom, the ones the users rated as not having. Unrated spheres would float up toward the top to vie for the users' attention and get him or her to rate them. Clicking on one of these spheres would then *open it up*, and the user would have the possibility to do exercises and register reflections and thoughts concerning the strength. Users are also easily able to add their own strengths and exercises should they want to.

Another key design decision from this workshop was to minimize the focus on the number of strengths a person has or the number of exercises done. This is because part of the rationale for a strengths focus posits that one uses strengths one already has [17]. As such, gaining new strengths is not a primary goal. To keep track of what users do in the app, we created the *daily log* section where each day's activity and input would be presented on cards listed onscreen. The log would additionally provide the users with the *three good things* exercise from positive psychology [10], daily asking the users for *three good things* they had experienced that day.

**Figure 7:**
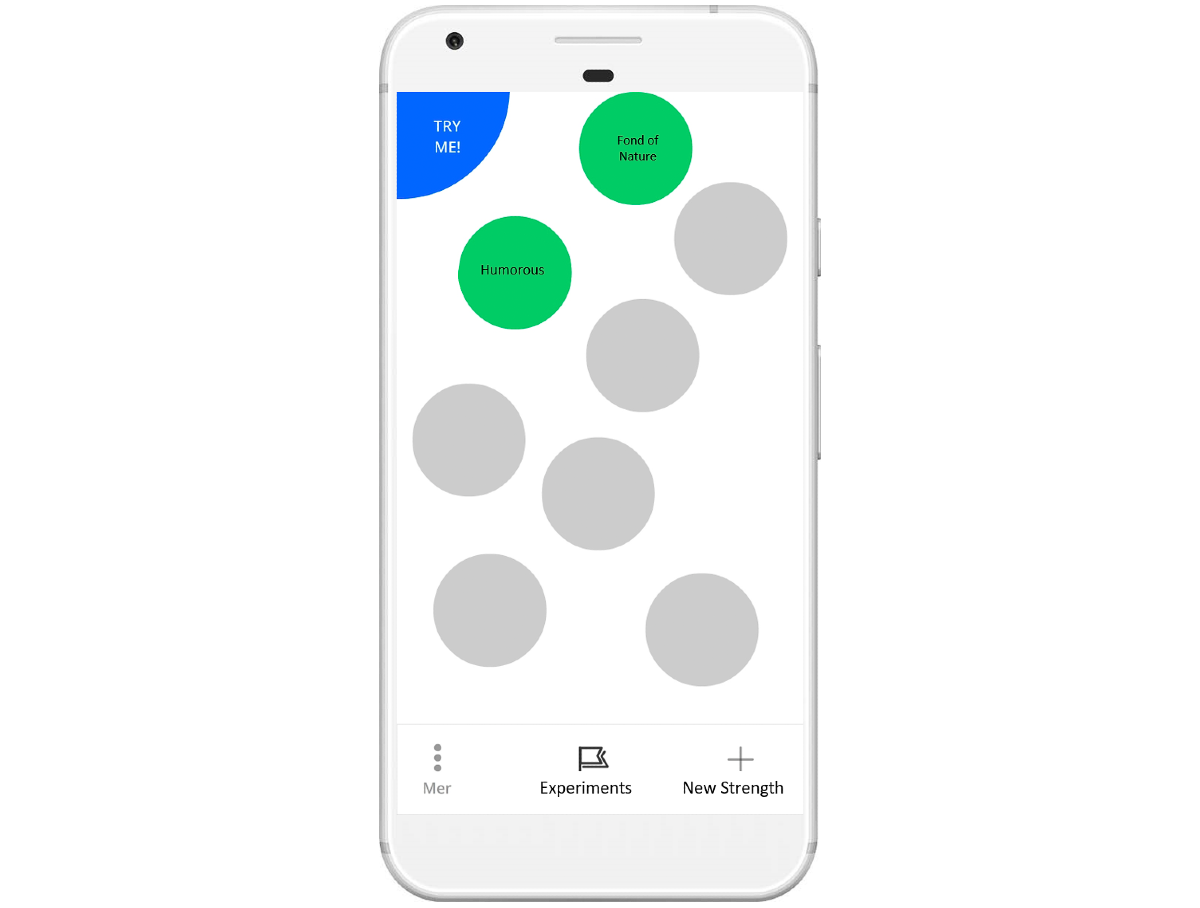
Home screen interactive mockup.

To engage users, time was spent discussing *novel* ways to interact with the app, and it was suggested that if the users shook the phone, this could, for example, result in the spheres on the screen moving around or prompting the users with a randomly suggested exercise. Although the *shaking idea* was eventually discarded because of both technical and design challenges, the concept of suggesting random exercises was refined through several iterations. Figure 7 presents a snapshot of an early interactive mock-up of the home screen with a *try me* button in the top left corner. Pushing the button, the app would then suggest an exercise for one of the users’ strengths at random. In the final design, this feature took inspiration from the world of games and the universally known roll of a dice, in what became the *dice* feature. In addition to surprising the users with randomly selected exercises, this randomness could possibly also provide users with new exercises very easily.

After several iterations of both paper-based and interactive mock-ups, our internal IT department started the development of the *MyStrengths* app based on the Unity [86] game engine.

###### User Evaluations

In 4 iterations, 13 users and 3 of our colleagues tried and evaluated the app. Table 3 presents the features added and evaluated, the number of participants, and their age and gender.

**Table 3:** Development iterations and users (N=13, 1 male).

Iteration number	User evaluations, n	Age (years), range	Internal testers, n	Features introduced in the current iteration
1	4 (1 male)	22-62	2	Introduction Pages Home screen Strengths assessment Strengths exercises (Marvel-app mock-ups)
2	4	20-50	0	Strengths exercises
3	3	51-59	1	Daily log
4	2	46	0	—^a^

^a^No new features added.

####### Overall

In sum, the feedback from the user evaluations was quite positive, and everyone reported to like the concept. However, the users also reported that the *daily log* section of the app was not particularly fun or engaging to use, and in a few cases, they even stated that they expected something *more fun* when clicking on it. Following this feedback, several of the participants contributed creatively with suggestions for improvements, and during the evaluations, they suggested adding a more game-like way to create goals and write entries in the daily log. Some of the evaluators also wanted to add pictures from the phone in the daily log. Due to all content created or added in the app being encrypted and stored locally, we could not add this functionality as it could possibly make the app’s data use on the devices enormous. Several participants suggested adding more visual flair overall, for instance, by using more animations and interactivity throughout or by adding avatars to represent you in the app. Many evaluators reported they were expecting to receive some notifications from the app, for instance, to remind them to do exercises or simply reminding them of their strengths. It was also suggested that the app in the notifications could ask users how they feel each day and then provide appropriate feedback, for instance, by cheering the user on if the day was good or reminding them of better times or their strengths if the day was bad.

####### Strengths

When trying the strengths experiments, several participants reported that they would instead work on gaining the strengths they were lacking than focusing on using the ones they had more. When asked about this, one participant explained, “Because this is how we are always taught to think.” Over the different iterations of the app, both the wording and style of the strengths exercises were subject to significant work and redesign. For instance, most users preferred exercises that were concise and simple to do (ie, not involving many steps of different actions needed). Along the same line, many users in the earlier iterations reported that some of the exercises were too complex and time consuming. Several users also commented on the impracticality of the strengths exercises asking them to write plans and thoughts down on paper, and they would instead prefer to do this on the phone itself. A large portion of the users also preferred exercises that were physical rather than cognitive or mental, such as “surprise a friend with something nice today” as opposed to “Sit down for 15 minutes and think about the good things you have in your life.”

####### Usability

**Figure 8:**
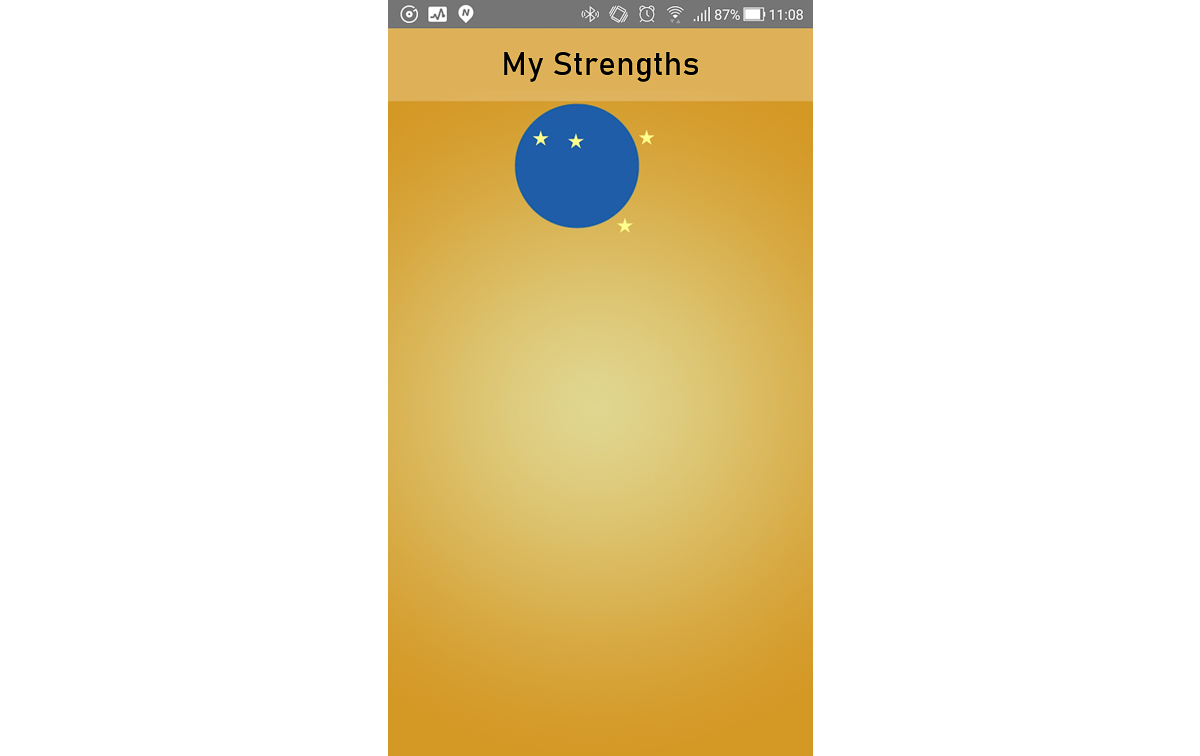
Screenshot of first strengths sphere.

In terms of user friendliness, we experienced during the evaluations that the intuitiveness of some sections the tool was not satisfactory. For instance, when the first sphere was shown on the screen, as shown in Figure 8, most participants did not click on or try to interact with it. This was redesigned, so that *unopened* spheres would pulsate to attract the users’ attention and indicate a possibility for interaction. Although we follow guidelines for universal design, a user with reduced dexterity still found some buttons difficult to hit. These were redesigned to be larger. We also encountered multiple instances of users not fully understanding icons and buttons. For example, on the page for performing strength exercises, one can choose between suggested exercises or add one’s own by pushing a button with a plus sign (+). During the evaluations, few of the evaluators used this button, even if prompted to add new exercises, and it was both redesigned and placed more prominently on the page.

There were also multiple technical issues discovered during the evaluations. In the earliest iterations, the app would freeze if users tried adding emojis as part of textual input. We also found an issue where the strengths spheres that should be *floating* to the top of the screen instead sank to the bottom of the available screen space. Overall, more than 120 design and usability issues as well as numerous bugs and technical problems in the *MyStrengths* tool were identified over the different iterations. The issues were redesigned and fixed between iterations.

####### Work With External Game Designers

**Figure 9:**
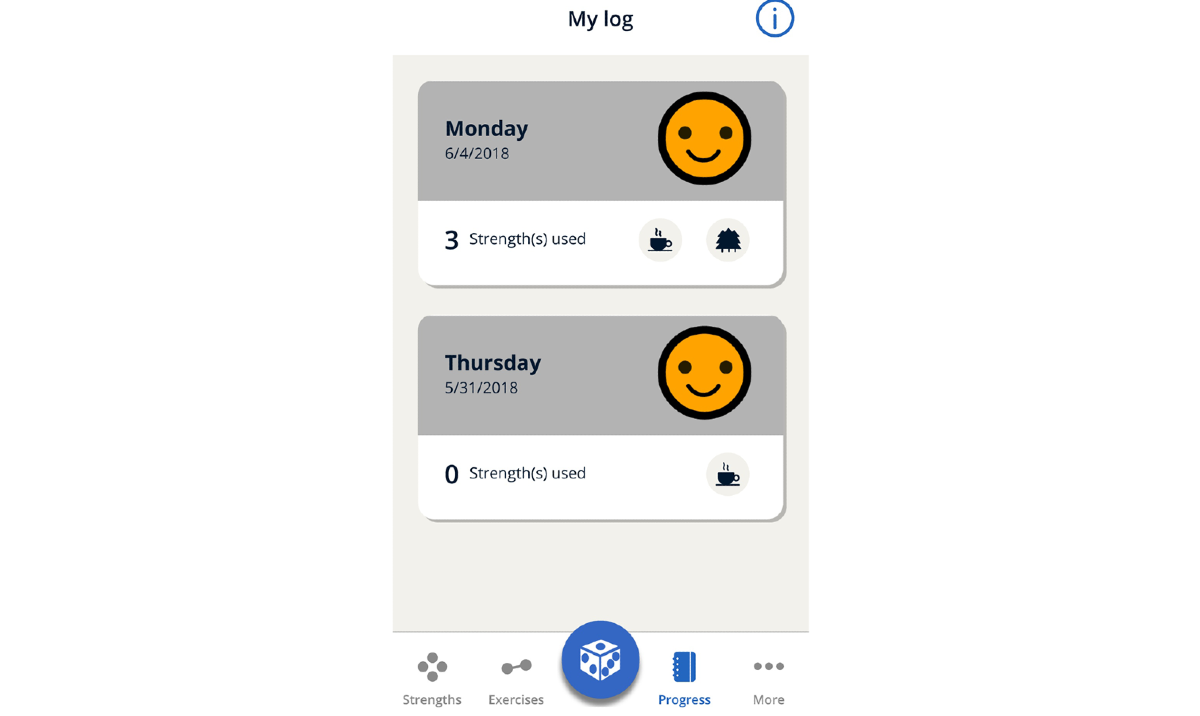
Daily log screenshot.

As feedback from user evaluations as well as the project group indicated a need for a more playful experience when using the *MyStrengths* app, we decided to consult with an external game designer to help expand on the gameful aspects of the daily log functionality, which at present visualized summaries of each day’s activities and registrations as *cards* (Figure 9).

**Figure 10:**
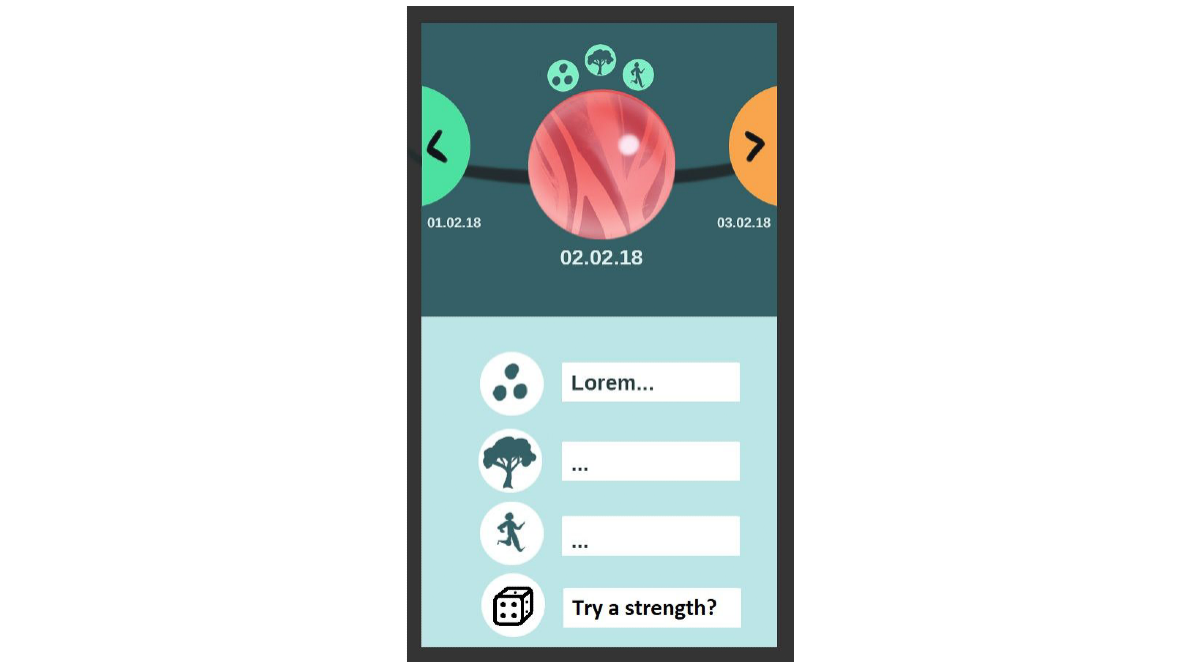
Daily log new concept sketch.

Working with the designer yielded a detailed concept description and rationale as well as mock-ups of the further developed daily log section of the app, an example of which is presented in Figure 10. The concept of spheres was also central to this, and entries into the daily log would look like pearls on a string one can swipe between. Like in the home screen, clicking on a sphere would *open it up* and show the details of that day’s entries. Hovering above each sphere would be small icons visualizing different activities registered during the day.

Although the project team found this concept to hold great potential, we were not able to implement these designs into the app because of both administrative and resource-related challenges as well as the need for the app to be ready in time for its feasibility trial. On the basis of the outcomes of this trial, we have planned to make adjustments to the app before making it generally available, and we aim to implement the new design concept during this period.

#### The Final *MyStrengths* App High-Fidelity Prototype

**Figure 11:**
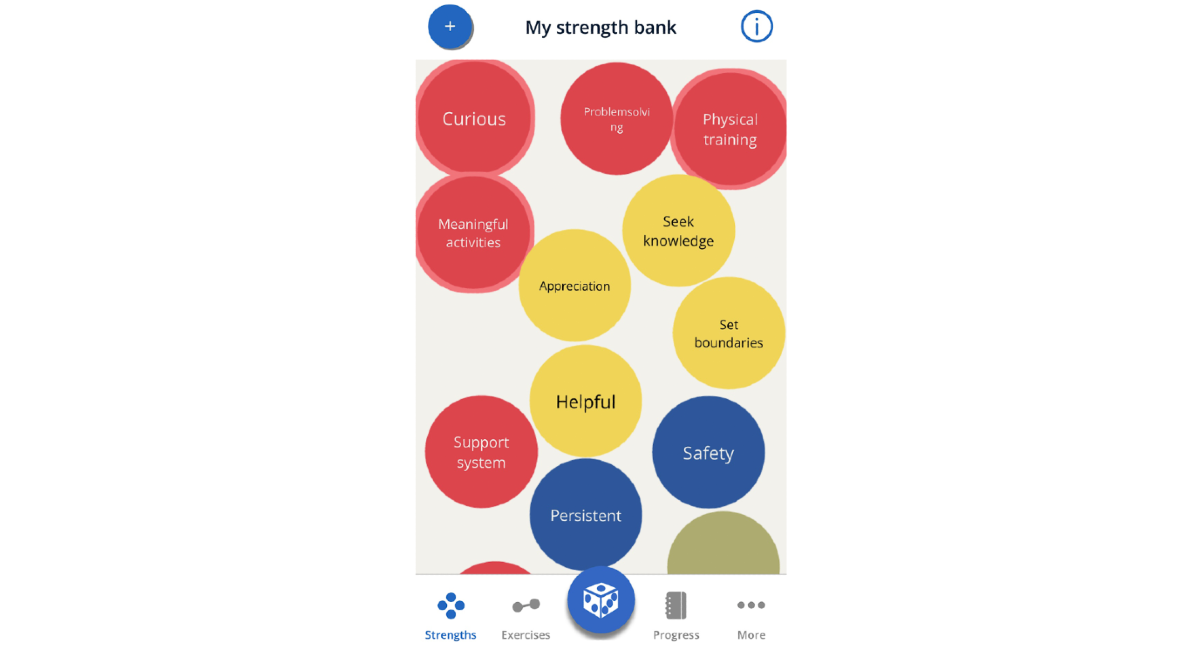
*MyStrengths* Home Screen.

**Figure 12:**
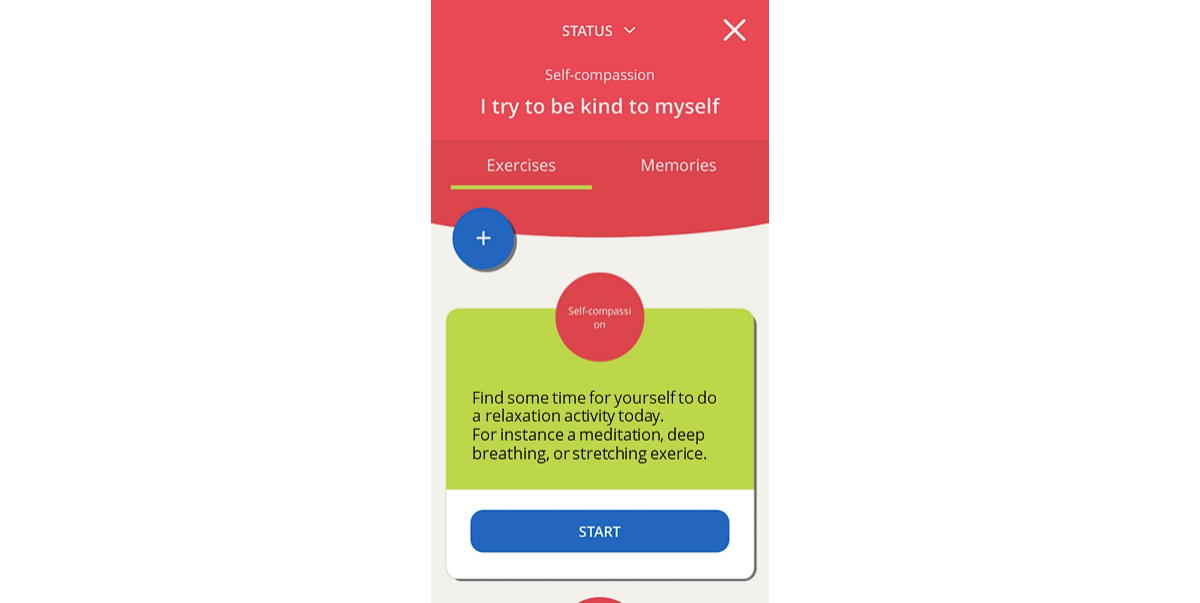
Strengths Exercise.

**Figure 13:**
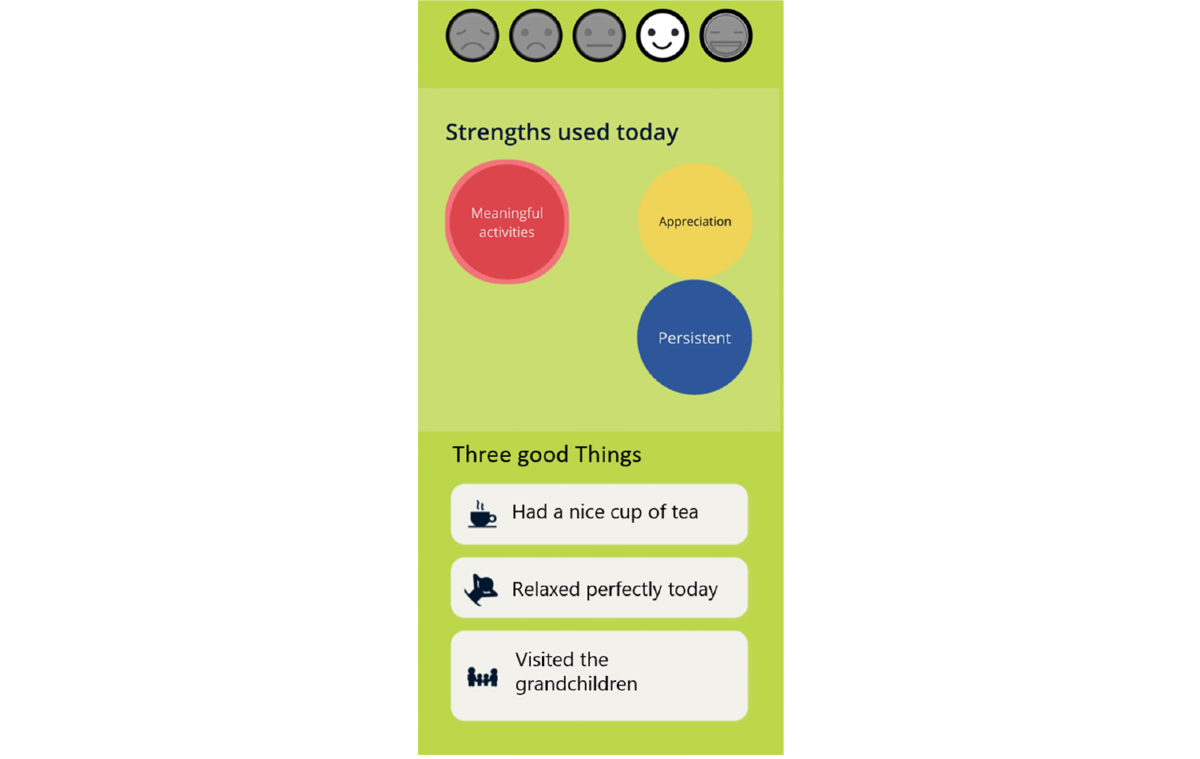
Rating the day in the daily log.

**Figure 14:**
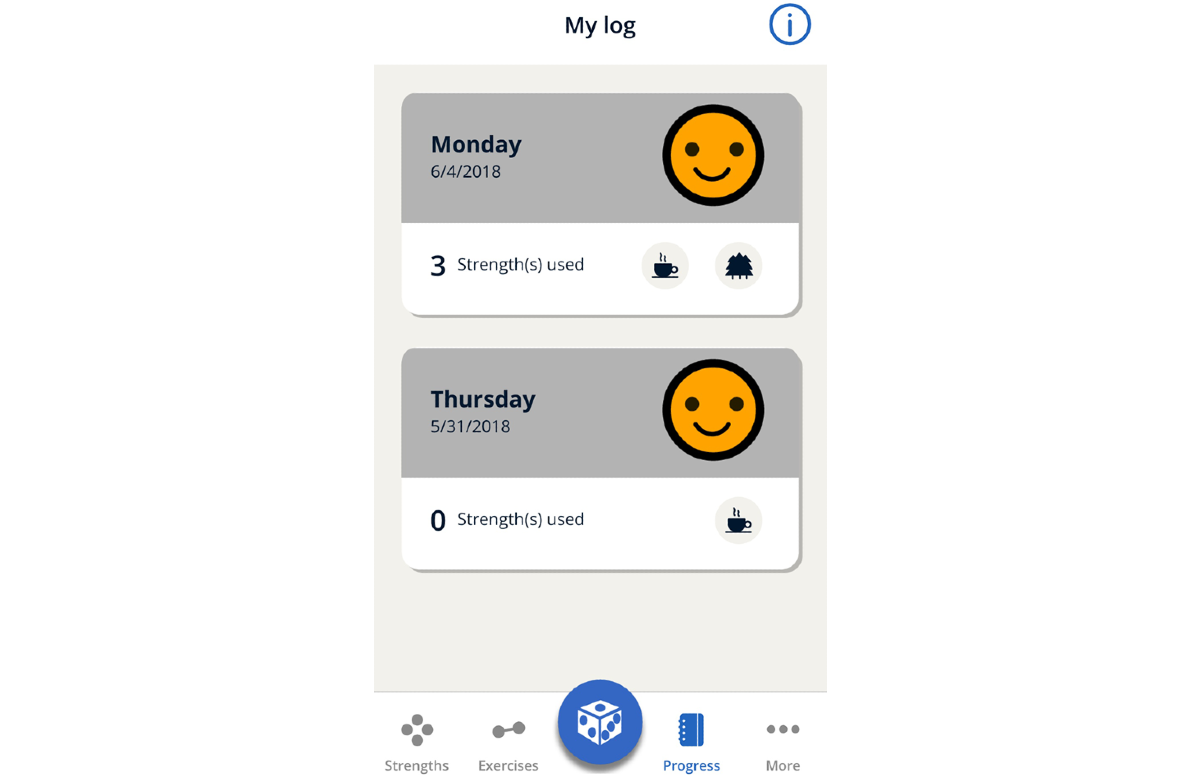
Daily Log.

**Figure 15:**
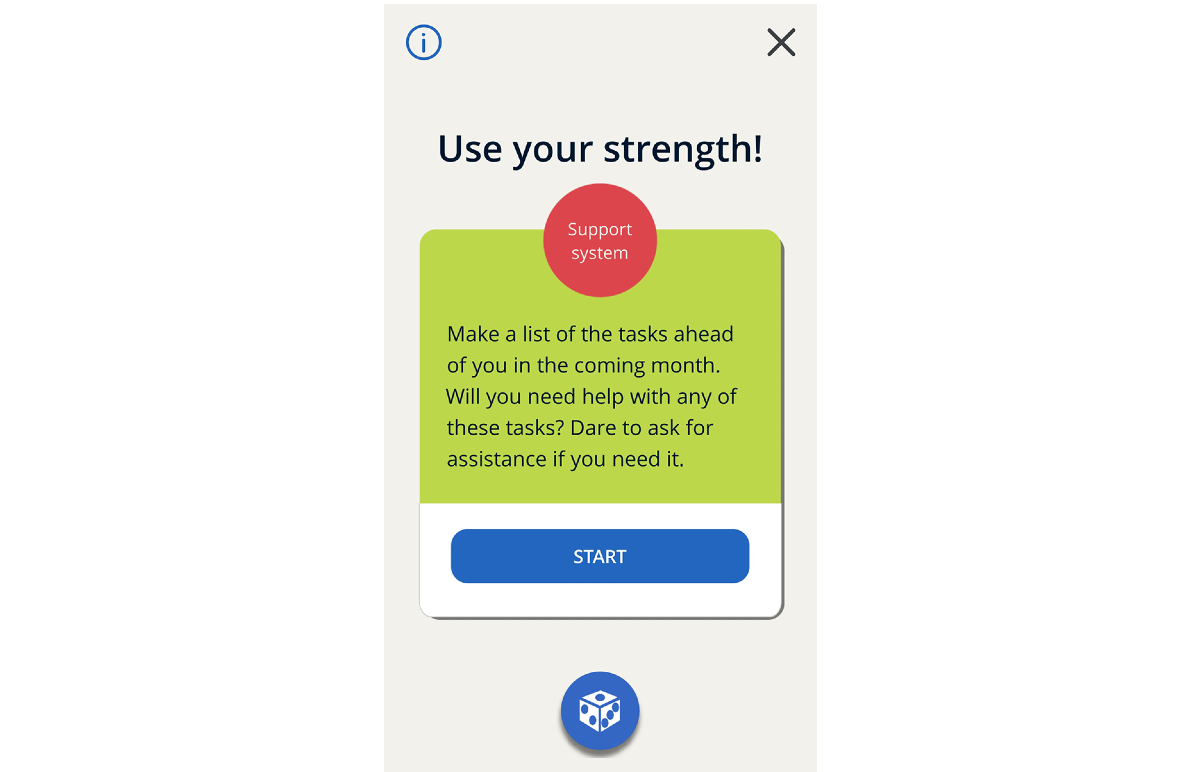
Dice Suggestion.

The finished high-fidelity prototype of the *MyStrengths* app centers on a list of strengths spheres floating around on the screen (Figure 11). These are colored red, yellow, blue, or green and signify which strengths one thinks one possesses, strengths one partly possess, do not possess, or have not yet assessed (or do not find relevant). Although the apps ask users to rate the 40 strengths, the user can also add as many new strengths as they please. From this screen, the user can access the 2 other key features of the app: strengths exercises and reflections and the daily log. Clicking on a sphere opens it up and provides the user with a list of suggestions for small exercises and the opportunity to create new exercises themselves or a note-taking area for writing small reflections on how this strength can help them in their lives (Figure 12). Active or completed exercises are listed in the exercises menu at the bottom of the screen. The daily log asks for an entry each day and when doing this first asks for a rating of how the day was, using 5 smiley faces (from sad to happy), then asks the person to write 3 good things that happened and pick icons for these (Figure 13). For each day, the log puts all this information into a summary and represents each daily entry as a card on a scrollable list (Figure 14). The dice at the bottom center of the screen randomly selects a strength exercise from the strengths that the user has and suggests exercises for the user to use this strength (Figure 15). A secondary goal of both the dice and the opportunity to add own strengths and exercises is that the app can provide users with new and interesting experiences, despite its basic set of features and content.

#### Privacy and Data Security

Maintaining user privacy is of high importance. As presented in phase 3, the privacy protection and data security committee at our institution has strict guidelines that led us to discard the interaction between users of the *MyStrengths* app. As such, users will not be able to identify one another. The app is planned to be available through both Google Play and the Apple App Store, and besides having an account in these stores, there is no need for the users to identify themselves or register before using the app. Although there are slight differences between data available to developers on Google Play and Apple App stores, the most detailed information on users available would be an aggregated number of users with different operative system versions. All data generated by users are encrypted and stored locally on the device. When launching the app for the first time, users create a four-digit pin code needed to access the app. A key for data encryption is generated, and this key is used to encrypt data before being stored. The encryption key itself is stored after being encrypted using a temporary key derived from the user's pin code. The app also conforms to the standards set by the European General Data Protection Regulation.

During a 4-week feasibility trial, the app will send usage data over a secure connection to the *Service for Sensitive Data* at the University of Oslo. Once the trial is completed, the registration and transmission of usage data will be disabled, and the app will only run locally on the users’ device. The feasibility trial has also been approved by the privacy protection and data security committee.

## Discussion

### Principal Findings

The availability of strengths-focused mHealth apps or guidelines for their design is scarce. This paper contributes to this field by presenting, in detail, the various activities, decisions, and outcomes from the design of the *MyStrengths* app. We have presented 3 types of findings stemming from the process of development:

1. From individuals in the target group who took part throughout, we have gotten both ideas and design requirements for strengths-focused mHealth tools. Chief among this input is the need to have such apps thoroughly focused on the user themselves and if using gameful designs to keep these mostly noncompetitive and positively oriented.
2. Having reviewed the existing literature on strengths-focused self-management interventions, we found a general lack of existing guidelines or design descriptions for strengths-focused mHealth tools. However, goal setting might be a productive way through which one might help people find and use more of their strengths.
3. Researchers and designers contributed, among other things, with knowledge and input on how strengths activities can be designed and connected to the users achieving set goals. This group also took part in prioritizing between, and merging, the various features and parts into the final *MyStrengths* app prototype.

From our experiences on this project, we would like to further discuss a few points and present some recommendations for others, creating strengths-focused and positive mHealth tools.

### Gameful Designs in a Positive and Strengths-Focused Environment

Recent literature reviews of gamefully designed eHealth and mHealth tools report the most popularly used game elements to all to be externally oriented: points, rewards, and leaderboards [36,37]. Still, through activities involving both users and experts, it was repeatedly suggested to be cautious with such elements, and during the co-design workshops, users voiced a specific dislike for designs with reward schemes that facilitate *addictive* use. This is similar to findings from the study by Ahtinen et al [46], where participants reported chasing rewards unfit for mindfulness exercises. However, in a review of apps promoting well-being and mindfulness [38], the authors found the archetypical game elements of points’ badges and competitions to continue to be the approaches mainly used.

Not overly using externally oriented motivational features, such as points and rewards, has also been discussed in more theoretical works on engaging and gameful designs [45,87,88]. These also highlight the importance of not only playing the game mechanics to win but to do the activities or tasks for the *right reasons*. This seems especially relevant for the *MyStrengths* tool, with its focus on the user’s reflections and awareness concerning themselves and their situation. As such, we decided to *tone down* the focus on gameful features that foster competition and aggregation of points or rewards and instead focus on engaging users with pleasant and positive user experiences.

Combining both social and goal-directed game elements into features such as competitions and collaborations is also very popular in gameful designs [37]. In addition, their potential for providing social comparisons and role modeling can make such features valuable in the field of mHealth [36]. However, users taking part reported concerns regarding visibly losing to, or receiving negative communications from, other users. To address this issue, the users during the co-design activities proposed ideas such as one-way communication using predefined texts or elements as a way of ensuring a positive focus. Anonymized social interaction or competition might be one way of leveraging the positive and motivational aspects of social interaction. This would also allow the user to avoid any perceived obligation of reciprocity or the possible stigmas of losing to one’s friends. An example of such an approach is presented by Mylonopoulou [89], who created leaderboards for progress in a mHealth tool, in which the user was shown and compared with 3 other anonymous users who were slightly better than them.

A gameful feature that, although not highly used, still holds potential is randomness [38]. During this project, we developed the *dice* feature that randomly selects strengths exercises for the users with the press of a button on the app’s home screen. The rationale for using randomness as a design element is that it can induce a sense of variety and anticipation of *what the future holds* for the user [38,90,91]. Randomness can be suitable for tools such as *MyStrengths*, in which there is no set route or user journey through the app. However, it is likely more challenging to include randomness in tools that are based on strict treatment regimens such as cognitive behavioral theory, in which the structure and order of activities is important [38]. The *dice* feature was well received by users in the evaluations, and we consider such approaches to hold great potential for providing gameful or playful experiences in an overall positive and gentle fashion. Thus, in technical terms, the design approach for this project can be described as focusing more on designing for *playfulness*, free play, and exploration, rather than *gamefulness*, which, in addition to free play and exploration, is more rule structured and often concerns a pursuit of points or scores [48].

### Strengths Approach to mHealth and eHealth

At the core of the strengths concept is changing the focus from deficits to the positive to achieve better well-being or happiness [14,17]. In keeping with this approach, emphasis should be placed on using existing strengths rather than focusing on turning non-strengths into actual strengths. Even so, during our user evaluations, several participants reported that they would instead work on gaining new strengths, as opposed to focusing on the ones they already have. This way of thinking is not that surprising and falls in line with the typical deficit focus of health care in general [92,93]. Therefore, future self-management tools using a positive or strengths approach should consider how to support users in shifting this way of thinking. In *MyStrengths*, the *dice* feature, which selects exercises at random, only draws exercises related to strengths that the user has assessed as having and thus guides them to build on their already identified strengths. This approach can be described as a way of nudging [94], a form of altering people’s behavior without forcing their options or activities. Furthermore, as an additional way of emphasizing the users’ existing strengths, the *MyStrengths* app sorts the strengths spheres so that the ones already marked as a possessed strength by the user are at the top and visible first when the app is opened.

Although assessment of people’s personal strengths has been done before (for instance, through tools or services such as *VIA Character strengths* [14] and *StrengthsFinder 2.0* [95]), these assessments have been made for the general population, not specifically for people living with chronic illnesses. This project, therefore, used a set of personal strengths that have been reported by, and found important for, people living with chronic illnesses [16]. Still, people’s strengths vary from person to person, and to improve the personal relevance of the tools for the users, we would also recommend to do as in the *MyStrengths* app and allow users to create their own strengths.

Despite previous research showing that the strength concept can be challenging for people to grasp, particularly when referring to one's own health [96], users participating in co-design activities as well as evaluations of the tool mostly understood the strengths concept quite well and were able to relate to it. One the other hand, users who took part in evaluating the first iterations of the app reported finding the strengths exercises difficult to grasp, and most of these participants also preferred the more physical and tangible exercises (such as “do something nice for a friend today”) over exercises that were either more cognitively challenging or consisted of many steps (such as “go to a library, borrow and read this book, then think how your journey as a patients likens the hero in the story”). On the basis of this type of feedback, the exercises were redesigned to be simpler to both understand and perform.

### Privacy and Ethical Considerations for Designing mHealth Tools

During all design phases in this project, users participating discussed privacy and the different ways in which the features of *MyStrengths* could affect this. For instance, during the co-design workshop, multiple participants talked about how one’s strengths were a very personal thing and something one might not want to share with anyone. Similarly, in the idea-generating workshop, several participants also voiced concerns about privacy and suggested using nicknames in communication with others. Clearly not wanting to share this information about themselves, one participant simply said, “I would never put something like this on Facebook” [78]. Participants in the user evaluations also highlighted concerns regarding privacy, and many would ask the facilitator whether anyone could view the information they entered into the *MyStrengths* app*.* As such, we can surmise that although people living with chronic illnesses, for instance, often are active in support and interest groups on social media [79], they should also likely be the ones to control what, if anything, to share.

As voiced by participants throughout this project, losing is no fun when it is owing to your own medical situation. Thus, it became important to make sure the *MyStrengths* app is respectful to users changing shapes, and we avoided using gameful design techniques such as awarding users for streaks, that is, using the app according to set schedules or intervals. Furthermore, when creating engaging, gameful, or persuasive designs, designers are steering users toward a desired action. In doing so, one should be careful not to steer users toward making choices that are against their interest or will or create a very limited set of perceived choices for interaction [97,98]. In *MyStrengths*, the *dice* only draws exercises for strengths that each person assesses themselves as having and will thus not suggest exercises for strengths that the user considers herself or himself not to have. In the *MyStrengths* app, this is explained to the users when going through the first-use tutorial of the app. However, users still have the possibility of selecting activities for all strengths in the app through the home screen. As such, it is important to consider the ethical aspects of mHealth tools. This can, as discussed above, mean not metaphorically *forcing the users hand* too much or using game design techniques that, for instance, are experienced as inappropriate, unfair, or even trivialize the user’s situation [45,47,90].

### Participatory Approach

In creating mHealth tools, it is highly beneficial for the design group to include researchers and/or designers with health, design, and psychology experience or background as well as actual representatives of the user group [51,52,97]. In this project, both the diverging phases started with involving users in a broad way to create ideas and suggestions before the converging phases relied more on evidence and input from researchers and designers to narrow the initial input down to concrete and implementable solutions. From their participation, we see that the users contributed valuable contributions such as (1) emphasizing a liking for a gameful design but not necessarily when it consists of stiff competition and possibly losing; (2) providing important input into the design and wording of the strengths exercises; and (3) how the *MyStrengths* app should fully be about the users, putting them at its center.

Although users did not actively take part in most of the converging phases, we were always focused on correctly representing their contributions and needs. In general, user inputs and concerns were maintained through 3 different strategies: (1) having a user representative, who is equal to everyone else in the project group (this person, it is important to highlight, works as a nurse and has a medical or nursing background, something that may also reduce the hierarchical distance between them and the researchers); (2) using the outcome from the co-design workshops and user evaluations in the discussions; and (3) having all project members and researchers do the same design tasks as the participants in the co-design workshop. This third strategy also supported the important task of communicating and creating a deeper understanding of the users’ needs and requirements within the project group [99]. This allowed the group to see not only the outcomes of the participants’ work but also to go through the process of creating similar types of work themselves.

In terms of the nonuser participants, a range of professions have been involved, including researchers with health, psychology, and informatics background and expertise; developers; designers; and game designers. Each of these participants contributed to broadening the group’s repertoire of possibilities with respect to the design of the *MyStrengths* tool. To name a few, (1) the psychology and health care researchers provided important information and feedback regarding the strengths approach, (2) the developers and designers contributed creatively in putting everything together into a working and user-friendly tool, and (3) involving the external game designer provided suggestions for more interactive and immersive solutions.

**Figure 16:**
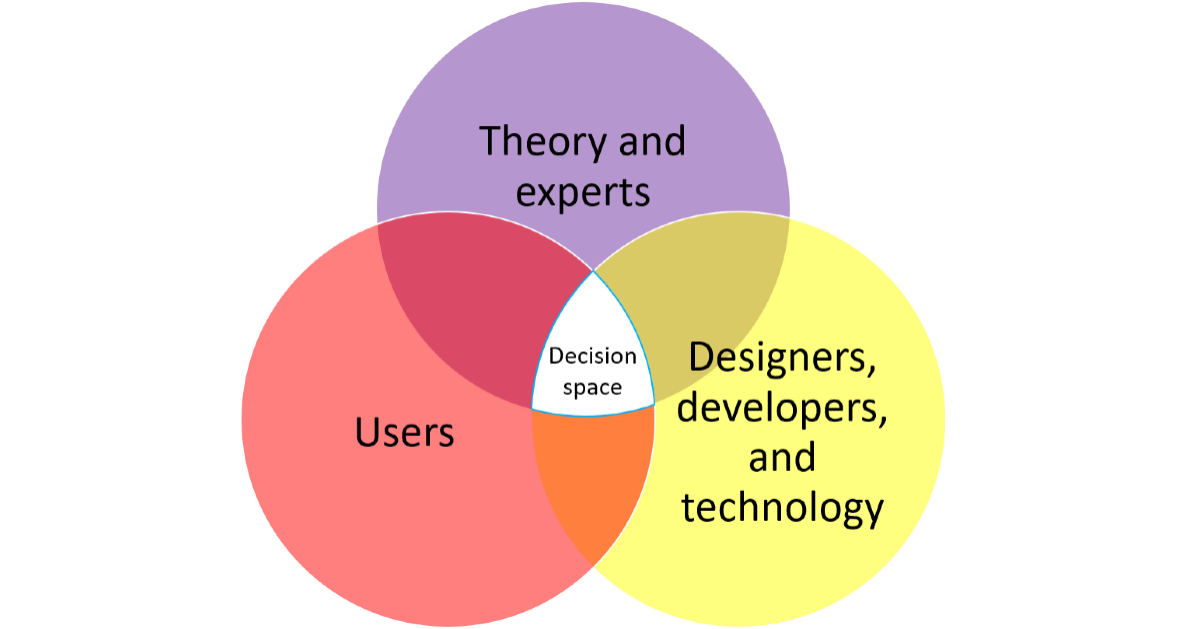
Venn diagram of decision space.

As such, the outcomes of this project can be considered to stem from a space between 3 different groups (see illustration in Figure 16): (1) theory, evidence, and researchers’ knowledge; (2) designers, developers, as well as the limitations and opportunities of the technology itself; and (3) the users, with their needs and ideas. Although the power of decision in this project was mostly placed with the researchers and experts, most decisions were the result of a negotiation between the 3 groups and their inputs into the project. Even though no users besides the user representative took part in the 2 converging phases, following the 3 strategies described above still allowed us to maintain and represent their needs, ideas, and requirements in the decision-making processes.

As shown by the range of outputs from the idea and design workshops, users are indeed capable of creating ideas and concepts with great potential. Although we thoroughly maintain the user’s inputs and ideas throughout the project, they are not able to contribute new input without taking part. This could, in particular, have changed the way in which we implemented and combined ideas and concepts stemming from the users in the first place. Still, users have been included and given the power and opportunity to create ideas or suggest ways to use the tool and to evaluate and suggest improvements to the design, the 2 aspects of a project that have the *strongest possibility for participation* [100]. As presented in this paper as well as in previous publications from this project [65,78], we have shown how the participants in our co-design activities contribute creatively and productively to both the content and design of the *MyStrengths* app.

### Lessons Learned From Creating *MyStrengths*

During the process of developing *MyStrengths*, we collaborated with a multitude of different stakeholders in a wide range of activities and processes. From these, we wish to highlight some points and issues that may be of interest to others developing positive or strengths-focused eHealth or mHealth tools for people living with chronic illnesses.

#### (Try to) Involve Users in All Activities

When projects clear the proverbial *fuzzy front end* [54] and finally start building the actual product, the pace of the work tends to increase. In earlier phases of the project, recruiting participants to activities was often done in collaboration with the institutions participating in the project's activities. By recruiting only for specific activities, we had to reach out again during later phases and re-recruit the same participants. This made organizing the recruitment and activities for participation time consuming and thus more challenging than envisioned. For instance, we would have benefited from additional design activities with users, both in connection with the designer workshop and in response to the user evaluations in phase 4. To achieve this, we could have recruited participants early in the project into a pool of available participants that we could easily contact to work more closely with as the project moved on. Although all design projects are highly dynamic, and schedules and priorities are often subject to unforeseen changes in resources, time, or technology, we would recommend creating a systematic plan for recruitment and participation for the entirety of the project.

#### Use User Representatives

Having a user representative as a member of the project group helped us in keeping touch with the users' perspectives and challenges throughout the work, even when the users were not participating. The user representative in our project was a full-fledged member of the group and took part in all activities and decision-making processes. For others employing representatives, we would also recommend including these as much as possible in activities and discussions to ensure they stay active and up to date and not end up passive sources, which the rest of the team only taps for information when needed. In addition, by the representative being a full member of the team, and not merely invited when needed, he or she gets to know the other project members better, and hierarchical differences in power are hopefully diminished.

#### Ideate Freely

We had to abandon the implementation of social features, primarily because of restrictions from the privacy officers at our institution. Although we could have foreseen this issue and removed the possibility of social features in our activities, we might have restricted the participants' creativity and range of possibilities. Furthermore, by working without restrictions during the workshops, participants also give important information and feedback that, for instance, are relevant not only to social features but also to mHealth tools as a whole.

It may seem more efficient concerning time and money to keep participatory activities focused on what is possible or advisable to create. However, we still recommend allowing for free creativity and ideation in such activities, as this can yield not only interesting ideas and concepts but also valuable insights into the user group and their needs and wishes.

#### Participatory Approaches in the Face of Incomplete Guidelines

Participatory approaches should be well suited in design situations where the aim is to create something new in the face of a shortage of guidelines for designing. This project aimed at both exploring ways of creating an engaging and strengths-focused mHealth app as well as actually developing one, *MyStrengths*. It is our position that those goals together make participatory approaches well suited. In addition, as our secondary goal is to explore opportunities for designing strengths-focused tools, it seems right indeed to include users, primarily as they should be considered to be experts of their own situation, and additionally as they can *widen the design space* [101] by, for instance, contributing ideas that the researchers and designers would not think of. Thus, users can also contribute productively in situations with solid evidence forming the basis of mHealth tools. As presented earlier, even how text is written and phrased benefited from user input. Thus, we would recommend considering all forms of creating mHealth tools, even small projects that, for instance, translate an existing tool, processes in which user participation can be of great value.

### Strengths and Limitations

The activities as well as outcomes presented in this paper can serve as a foundation for future research on the development of strengths-focused mHealth or eHealth interventions for people living with chronic illnesses. It is important to note that because of the explorative nature of this project, any generalizations as to what designs or functionality people living with chronic illnesses enjoy or want is neither possible nor intended. However, based on both the quality and range of output presented in this paper, it is likely that our chosen activities and methods worked well and may be applicable to others as well.

However, there are also some limitations to this study. First, all users and stakeholders participating in the study volunteered to participate on their own or were contacted by the project team or collaborating institutions. As such, the participating groups are likely biased toward being more motivated, resourceful, and managing their life with chronic illness well. Thus, they may not perfectly represent the entire user group. However, this is too common for this kind of research. Using other means for recruitment, such as social media [102], might have eased access to harder-to-reach users, but this was not within the project's mandate.

Second, by aiming to design a mHealth tool for such a broad group (ie, people aged >16 years living with chronic illness), this project sought to design for a target group that is practically the entire population. However, creating features for specific or smaller groups was not the goal of the project, among others, as having strengths is something shared by everyone irrespective of illnesses, age, or background.

Third, the gender balance during most of the project activities was skewed toward female participants, with around only one-third of the participants being male. During the user evaluations, only one of the participants was male. During all recruiting, we tried to recruit more male participants, and unfortunately, we were never able to reach equal number of males and females. Similarly, both age and gender distribution among the participants changed throughout the project. However, having participants from different age groups also means that they represent the age range of the intended user group.

Fourth, regulations concerning privacy and data security made us decide to drop the inclusion of social functionalities in the *MyStrengths* app. Although necessary for us to do, this also meant that we discarded one of the more popular features suggested by participants throughout all our activities. Similarly, because of unforeseen challenges at our end as well as for our external collaborators, we were never able to implement the redesign of the daily log part of the app.

As it stands, some of the features and concepts suggested by users, designers, or researchers throughout this project for various reasons ended up unused. Technical challenges and restrictions or overruns on time and resources are not uncommon in these kinds of projects. Many of the technical challenges faced are a product of us trying out new designs or forms of interactions, such as the strengths spheres floating on the home screen or the initial idea of shaking the device to move spheres around. Although time consuming, this experimentation is also in line with the projects’ goal of exploring new ways of creating strengths-focused and engaging mHealth tools. The design of strengths-focused mHealth or eHealth interventions has rarely been reported, and both the activities and outcomes reported in this paper add important new information to this growing field.

We also wish to highlight the fact that the *MyStrengths* app was developed in a very collaborative way with members of the users and stakeholder groups. In fact, 2 of the participants took part in all activities with user involvement, idea workshops, co-design workshops, and user evaluations. Although caregivers only participated in the first phase, their perspective was not lost in the later phases, as several members of the project team have backgrounds in nursing and health promotion work. The repeated involvement of many users is an excellent strength to the project. It allows researchers, developers, and participants to create a thorough and deep understanding of each other and the tools being designed.

Everyone has their own strengths [14,17], and as such, the *MyStrengths* app has great potential in helping the increasingly large group of people living with chronic illnesses. Although the *MyStrengths* app holds great promise, we cannot, at present, speak to the effects or benefits of its use. However, a real-world feasibility trial with the *MyStrengths* app is currently ongoing, the results of which will be presented in a future publication.

### Conclusions

Supporting people living with chronic illnesses in focusing on their strengths and positive resources can lead to higher well-being and quality of life. This project developed the *MyStrengths* app, a tool supporting its users to use their strengths more actively in their everyday life. As there currently exists little guidelines on developing mHealth tools with a strengths focus, this project took a participatory approach to create an engaging and playful mobile app to address this gap in research. During this process, we explored a range of ways of implementing strengths into mHealth tools and how to make such tools more engaging for its users. Although participatory design projects take time and are resource intensive, designing a tool such as the *MyStrengths* app based solely on the knowledge and ideas from literature and researchers is likely not a sound strategy as the user perspective is often lacking. In this project, we have shown how users can contribute productively to ensure that mHealth and eHealth tools being developed are both accepted and understood well.

Adding to the growing field of designing strengths-focused mHealth tools, this paper presents our approach for creating the *MyStrengths* app, which is based on evidence and established theories as well as created with contemporary design methods and with a high degree of user participation. Outcomes from, and methods used during, this project can be used as a starting point for future studies exploring strengths or strengths based on mHealth and eHealth tools. We kindly invite others to further develop, adapt, and build on findings, ideas, and activities presented in this paper for their own contexts.

## Appendix

Multimedia Appendix 1 Brief overview of review findings.

## Abbreviations

eHealth: electronic health
mHealth: mobile health
IT: information technology
