# Supplementary material for: MyStrengths, a Strengths-Focused Mobile Health Tool: Participatory Design and Development
Source: JMIR Form Res. 2020 Jul 24;4(7):e18049. doi: 10.2196/18049 (PMC7414410; doi:10.2196/18049)
Supplement: Multimedia Appendix 1 [file formative_v4i7e18049_app1.docx]

# Appendix 1, Brief overview of review findings

| **Paper** | **Strengths activities and delivery** | **Positive outcomes on** |
| --- | --- | --- |
| Cerezo and colleagues (2014) | 14 two-hour weekly in-person sessions with 3 modules of positive psychology activities  Module 1   - Self-knowledge module: promoted psycho-emotional introspection skills, i.e., self-knowledge and self-awareness   Module 2   - Positive psychology related skills: promoted coping styles using positive psychology related skills, positive based behavioral and emotional activation   Module 3   - Trait-related coping skills: Promoted enhancing psychological strengths and applying skills derived from psychological strengths towards adequate coping in difficult situations | Wellbeing  Positive emotions  Emotional processing, optimism, resilience, hope  Negative affect |
| Cheung et al. (2017) | 5 weekly 1 hour session with two intervention groups:   - In-person, with a trained facilitator - Online, self-paced modules with new lessons available seven days after the previous   Teaching empirically based skills designed to increase the frequency of positive emotions, including:   - noticing positive events - gratitude - focusing on personal strengths - setting and working toward attainable goals - small acts of kindness | Psychological wellbeing:   - Depression - positive and negative emotion - cancer-specific quality of life   Positive coping:   - Mindfulness - Positive-affect skill use - Self-compassion |
| Cohn et al.(2014) | Self-paced online intervention consisting of 5 weeks one-hour sessions, one week with strengths focus  Week 4   - Strength related skill development: Learning how to recognize personal strengths and acknowledge them even when they are feeling bad about oneself - Strength exercise: Daily strengths journal where you record ways you used a personal strength or talent | Depressive symptoms |
| Nikrahan et. al (2016) | In-person group training program consisting of 6 weekly 90-minute sessions.  3 intervention groups with a range of positive psychology activities, including:   - Identifying a signature strength from a list of 24 personal traits - Use a signature strength in daily activities - Identify strengths in their partners and children - Use a signature strength in a way that futures a cause larger than oneself - Use a signature strength in a way that futures a cause larger than oneself - Focusing on positive personal traits - Savoring life’s joys - Fostering self-knowledge and self-esteem - Forgiveness   - Setting aside negative feelings | Happiness and hope |
| Muller et al. (2016) | Participants received email with pdf containing instructions to perform at least one strengths exercise weekly for 8 weeks.  Each participant received a list of four exercises tailored to their interests, based on Person-Activity Fit Diagnostic tool. Pool of exercises included a focus on:   - Kindness - Gratitude - Savoring - Taking care of the body - Spirituality - Relationships - Goals - Forgiveness | - At post treatment intervention resulted in a statistically significant increase in life satisfaction, positive affect, and pain control, and a significant reduction in depressive symptoms, pain intensity, pain interference,   and pain catastrophizing.   - Positive effect on pain intensity and pain control post treatment, but not at a 2.5-month follow-up. - At the 2.5-month follow-up: improvements in life satisfaction, depressive symptoms, pain intensity, pain interference, and pain control were maintained for the intervention group. |
| Zangi et al.(2012) | 10 4,5-hour long group sessions over 15 weeks, two sessions focused on strengths.  Session 2   - “Who am I? - My personal resources”   Session 5   - “What do I need? - Knowing one’s strengths and limitations | Self-care ability  Wellbeing  Emotional processing  Psychological distress |

## References

Cerezo MV, Ortiz-Tallo M, Cardenal V, de la Torre-Luque A. Positive Psychology Group Intervention for Breast Cancer Patients: A Randomised Trial. Psychol Rep 2014;115(1):44–64. PMID:25153949

Cheung EO, Cohn MA, Dunn LB, Melisko ME, Morgan S, Penedo FJ, Salsman JM, Shumay DM, Moskowitz JT. A randomized pilot trial of a positive affect skill intervention (lessons in linking affect and coping) for women with metastatic breast cancer. Psychooncology 2017;26(12):2101–2108. PMID:27862646

Cohn MA, Pietrucha ME, Saslow LR, Hult JR, Moskowitz JT. An online positive affect skills intervention reduces depression in adults with type 2 diabetes. J Posit Psychol 2014;9(6):523–534. PMID:25214877

Nikrahan GR, Suarez L, Asgari K, Beach SR, Celano CM, Kalantari M, Abedi MR, Etesampour A, Abbas R, Huffman JC. Positive Psychology Interventions for Patients With Heart Disease: A Preliminary Randomized Trial. Psychosomatics 2016;57(4):348–358. PMID:27137709

Nikrahan GR, Laferton JAC, Asgari K, Kalantari M, Abedi MR, Etesampour A, Rezaei A, Suarez L, Huffman JC. Effects of Positive Psychology Interventions on Risk Biomarkers in Coronary Patients: A Randomized, Wait-List Controlled Pilot Trial. Psychosomatics 2016;57(4):359–368. PMID:27137709

Müller R, Gertz KJ, Molton IR, Terrill AL, Bombardier CH, Ehde DM, Jensen MP. Effects of a Tailored Positive Psychology Intervention on Well-Being and Pain in Individuals with Chronic Pain and a Physical Disability. Clin J Pain 2016;32(1):32–44. PMID:25724020

Zangi HA, Mowinckel P, Finset A, Eriksson LR, Hoystad TO, Lunde AK, Hagen KB. A mindfulness-based group intervention to reduce psychological distress and fatigue in patients with inflammatory rheumatic joint diseases: a randomised controlled trial. Ann Rheum Dis 2012;71(6):911–917. PMID:22186709
